# A GAN-Based Approach for enhancing security in satellite based IoT networks using MPI enabled HPC

**DOI:** 10.1371/journal.pone.0331019

**Published:** 2025-09-25

**Authors:** Syed Zubair Ahmad, Farhan Qamar, Hamdan Alshehri, Fathe Jeribi, Ali Tahir, Shams Tabrez Siddiqui, Jayabrabu Ramakrishnan

**Affiliations:** 1 Computer Engineering Department, University of Engineering and Technology, Taxila, Punjab, Pakistan; 2 College of Computer Science and Information Technology, Jazan University, Jazan, Saudi Arabia; Cardiff Metropolitan University - Llandaff Campus: Cardiff Metropolitan University, UNITED KINGDOM OF GREAT BRITAIN AND NORTHERN IRELAND

## Abstract

Satellite Internet of Things (IoT) networks based on satellites are becoming increasingly critical for mission-critical applications, including disaster recovery, environmental surveillance, and remote sensing. While becoming more widespread, they are also more vulnerable to various risks, particularly due to the heterogeneous communication technologies they support and the limited computing capacity on each device. When such IoT systems are connected with central HighPerformance Computing (HPC) clouds, particularly by satellite links, new security issues arise, the primary one being the secure transmission of confidential information. To overcome such challenges, this research proposes a new security framework termed DLGAN (Deep Learning-based Generative Adversarial Network), specially designed for satellite-based IoT scenarios. The model leverages the strengths of Convolutional Neural Networks (CNNs) for real-time anomaly detection, combined with Generative Adversarial Networks (GANs) to generate realistic synthetic attack data, thereby addressing the challenge of skewed datasets prevalent in cybersecurity research. Since training GANs may be computationally expensive, the model is optimized to run on an HPC system via the Message Passing Interface (MPI) to enable scalable parallel processing of huge IoT data. Fundamentally, the DLGAN model is based on a generator/discriminator mechanism for effectively distinguishing network traffic as either benign or malicious, with the capability to detect 14 different types of attacks. By harnessingAI-enabled GPUs in the HPC cloud, the system can provide fast and accurate detection while maintaining low computational costs. Experimental evaluations demonstrate that the framework significantly enhances detection accuracy, reduces training time, and scales well with large data volumes, making it highly suitable for real-time security operations. In total, this study highlights how integrating advanced deep learning technologies with HPC-based distributed environments can deliver an efficient and dynamic defense mechanism for contemporary IoT networks. The envisaged solution is unique in its ability to scale, maximize efficiency, and resist attacks while securing satellite-based IoT infrastructures.

## Introduction

**Fig 1:**
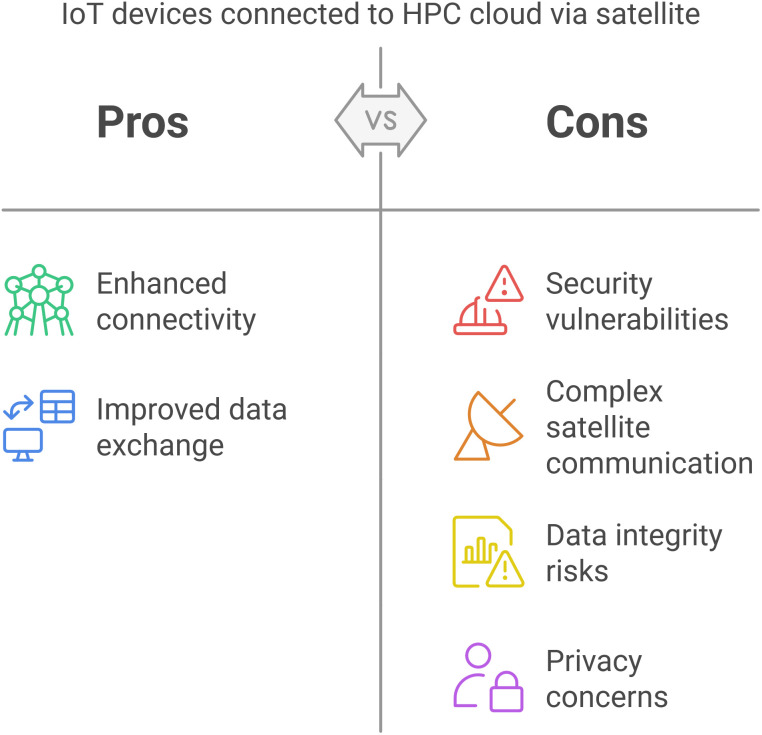
Pros and Cons of IoT Sensors Communicating to an HPC enabled Cloud Using Satellite.

The ubiquitous installation of IoT devices and sensors in various industries has created a need for global connectivity and real-time data sharing. Such increased interconnectivity, however, poses an enormous security threat, especially when IoT devices interact with central cloud infrastructures through satellite communications. This paper examines the compounded security issues in such setups, particularly the exacerbated vulnerabilities resulting from both the intrinsic limitations of IoT devices and the complexity of satellite communication. The security vulnerabilities inherent in IoT nodes when they are integrated with satellite-based communications have significant implications for data integrity, confidentiality, and system resiliency. Fig 1 lists the relevant advantages and disadvantages of the architecture. Despite noted drawbacks, embedding IoT devices in HPC cloud environments offers a promising remedy to enhance computational efficacy and facilitate real-time data processing.However, end-to-end security must still be guaranteed, mainly when satellite communication links are used. Satellite links are vulnerable to various attacks, including signal interception, jamming, and spoofing, which compromise the confidentiality and integrity of the data transmitted and the overall security of the system. Additionally, since IoT devices often have limited computational power, they are not generally able to maintain sophisticated security protocols, making them particularly susceptible to cyberattacks.

This section summarizes the widespread vulnerabilities of IoT sensors and devices, situating them within the broader context of satellite-connected HPC-embedded infrastructures and highlighting the pressing need for practical, scalable security solutions.

### Weaknesses of IoT devices

#### Limited security features.

IoT sensors and devices are often designed with limited in-built security features, typically featuring weak encryption protocols and authentication methods. This lack of functionality makes them vulnerable to unauthorized use and exploitation, hence creating a serious threat to the integrity and confidentiality of the entire system.

#### Insecure communication protocols.

The application of outdated or insecure communication protocols also significantly increases the susceptibility of IoT devices to a wide range of security threats. The most common among these threats is the Man-in-the-Middle (MITM) attack, where an attacker intercepts data exchanged between devices and gains unauthorized access, potentially tampering with or altering the communication stream.

#### Physical security risks.

IoT devices are often installed in remote or inaccessible areas, subjecting them to a multitude of cybersecurity (CS) threats, ranging from physical tampering to theft. These physical loopholes could potentially be the point of entry through which attackers can gain access to the underlying system. The IoT ecosystem is growing exponentially, and interrelated devices, such as sensors, actuators, smart homes, and smart city infrastructure, are the pillars of IoT-enabled industrial transformation.

Besides the above vulnerabilities, IoT networks produce enormous amounts of data through highly connected devices. This data needs to be processed efficiently, typically by HPC based cloud infrastructures [1]. The advent of satellite internet services, such as those provided by SpaceX and other similar players [2], has brought satellite communication within reach. Today, it is being widely utilized not just to link IoT devices to cloud servers but also among the distributed nodes of HPC infrastructure.

Although satellite communication provides connectivity in geographically inaccessible or infrastructure-scarce areas, it also comes with new security threats. The use of satellite links in such implementations increases the vulnerability of IoT devices to cyberattacks, particularly due to the inherent vulnerabilities associated with satellite communication protocols. Consequently, the increasing interconnection of IoT with satellite networks has raised concerns regarding data confidentiality, integrity, and system resilience.

To address these challenges, various cybersecurity measures have been proposed [3]. Among them, Intrusion Detection Systems (IDSs) are critical components for detecting security incidents within IoT environments and maintaining the integrity of secure and consistent services [4,5]. Implementing effective IDSs in IoT settings can be challenging. The limited processing capacities of IoT devices, combined with the vast amount of data generated, make real-time intrusion detection more difficult. These restrictions pose significant impediments to the successful rollout of traditional IDS models.

Additionally, in most remote deployments, constrained bandwidth and connectivity render impractical the process of forwarding extensive data sets to central cloud servers for processing [6,7]. In such cases, satellite communication presents itself as a feasible, but insecure, option, requiring strong and adaptive intrusion detection mechanisms, particularly suited to satellite-facilitated IoT networks.

To address the security issues observed in satellite-connected Internet of Things (IoT) networks [7], we propose an Intrusion Detection (ID) method based on a Deep Learning Generative Adversarial Network (DLGAN). This new method utilizes effective and adaptive intrusion detection mechanisms to counteract the processing resource constraints inherent in IoT devices and sensors. Our primary goal is to improve the security posture of IoT infrastructure deployed with satellite-linked infrastructure by integrating DLGAN into a high-throughput, scalable framework.

The proposed DLGAN framework serves a twofold function: it enhances anomaly detection and generates new synthetic data samples to mitigate dataset imbalance. It is customized for the cybersecurity needs of satellite-connected IoT devices linked to a central cloud, backed by a Message Passing Interface (MPI)-based HPC infrastructure. Using the dataset mentioned in [4], we create an architecture that aggregates data from geographically dispersed IoT nodes and sends it to a central HPC cloud.

This cloud infrastructure comprises MPI-driven HPC servers distributed across various regions. The servers are equipped with GPU-enhanced cores that can run deep learning models with the aid of frameworks such as Keras and TensorFlow. The collective data is processed in real-time, allowing the system to detect intrusions and label traffic as either benign or malicious.

The experimental configuration assesses the DLGAN model’s performance under different setups. Specifically, we investigate the impact of model hyperparameters on detection precision, computational cost, and processing time. Our solution demonstrates that real-time, scalable IDS can be effectively deployed in satellite-enabled IoT networks utilizing distributed HPC resources.

Advanced deep learning (DL) methodologies, when combined with HPC architectures, constitute the building blocks of our proposed Deep Learning-based Generative Adversarial Network (DLGAN), whose purpose is to tackle some of the most important security issues in satellite-based IoT networks. DLGAN functions based on double neural network architecture, comprising a generator and a discriminator, trained adversarially to produce synthetic data that closely resembles actual traffic patterns. These models are based on security-augmented adversarial training protocols, specifically designed for the intricacies of satellite communication systems [8,9]. The increasing use of IoT devices, especially in satellite-based environments where sensitive information is sent over open-air channels, has heightened cybersecurity threats. This has motivated stepped-up research attention towards the application of DL-based models like GANs for intrusion detection and anomaly inspection [10,11].

Our proposed DLGAN approach is particularly advantageous due to its integration with MPI-capable HPC servers, which significantly enhances data processing capacity and scalability.

Such integration not only enables advanced anomaly detection mechanisms but also supports better threat modeling and mitigation techniques. The large, imbalanced dataset considered in our study represents real-world IoT environments and poses challenges common in real-time analytics applications [12,13]. Here, DLGAN is found to be effective in generating synthetic samples from the minority class in imbalanced datasets. Such capability enhances security algorithm training and subsequent improvement in the accuracy of attack detection and the feasibility of preventive measures in a time-efficient manner [14,15]. However, although the inclusion of DLGANs in satellite IoT networks provides much added security and performance, it comes at a cost. Computational complexity, model stability in terms of convergence, and deployment overhead are areas that require significant attention to fully realize the potential of such robust architectures in real-world deployments.

The widespread use of satellite communication in IoT networks is primarily limited by the prohibitive costs of operations and the complexities associated with adopting sophisticated technologies [11]. Moreover, the dynamic and fluid nature of the security threat environment requires DLGAN-based systems to evolve to counter new attack patterns. This also highlights the need for ongoing research and development to enhance their real-time detection capabilities, as well as to provide uncompromising protection of sensitive information in satellite-based IoT infrastructures [16,17].

In summary, the combination of deep learning, satellite communication, and IoT security using DLGANs is a promising way to improve the cybersecurity resilience of future IoT architectures.

Yet, to make robust and adaptive defense systems a reality, model scalability, attack generalization, and deployment efficiency will need to be advanced. Solving the security issues related to satellite communication, more specifically, ensuring the integrity of data in distributed IoT settings, whichrequires ongoing innovation and inter-disciplinary research in the areas of artificial intelligence, network security, and distributed computing [18,19].

### MPI-enabled HPC architecture

HPC constitutes the core of the proposed architecture, playing a central role in processing massive datasets and performing computationally demanding operations with maximum efficiency. This is especially important in applicationsinvolving Deep Learning (DL)-based Generative Adversarial Networks (GANs), which are used to enhance the security infrastructure of satellite-enabled IoT networks.

A typical HPC setup consists of Graphics Processing Units (GPUs) and connected nodes facilitated by the Message Passing Interface (MPI). These nodes work together to provide task-level parallelism, where individual nodes perform calculations independently while ensuring synchronization and communication with other nodes to provide a harmonized and uniform output [8]. In our framework, this uniform output is equivalent to creating realistic synthetic data samples for training the Intrusion Detection System.

#### Core components of MPI-enabled HPC architecture.

The Message Passing Interface (MPI) is a fundamental component of HPC architectures, enabling efficient communication and parallel computing among distributed nodes within a computing cluster. For DL-based GAN systems, MPI is crucial for coordinating computations across different nodes. In particular, our design utilizes MPI4Py, a Python implementation of MPI, to efficiently handle inter-node communication and task scheduling. Through the use of MPI4Py, the system realizes improved performance, scalability, and computational efficacy, vital for processing massive datasets and training sophisticated DL models in real-time [12].

#### Mandatory components.

Compute Nodes (Servers/Workstations): Compute nodes are the fundamental components of an HPC architecture, serving as the primary processors for running computationally intensive tasks. Each node is typically equipped with high-end hardware components, including 4th Generation Intel Xeon Scalable Processors and GPU chips that feature integrated Tensor and CUDA cores. These hardware accelerators are designed with high core density and specialized support for advanced software frameworks, enabling the effective parallel processing of large deep learning workloads. In our architecture, these compute nodes serve a key function in training and running GAN-based intrusion detection models, both speed and scalability [12].

#### Storage (HDD, SSD, NVME, GPU memory).

The storage subsystem of an HPC system is crucial for handling the large amounts of data generated and processed during deep learning operations. Intermediate results, training data, and computation-intensive output are stored by it. High-read/write-throughput storage is essential for providing rapid data access, low latency, and uninterrupted data flow within the system. High-speed storage devices, such as GPU-attached storage and system-level SSDs, are critical performance-enabling technologies in our proposed IoT architecture, enabling high-throughput capabilities throughout the cluster to support real-time analytics and model training workflows.

A scalable and high-performance networking system is critical in enabling efficient communication between compute nodes and GPUs in an HPC cluster. Efficiently optimized networking has a vitalimpact on the system’s capability to process large-scale, real-time applications, such as deep learning-based security monitoring in IoT networks enabled by satellites. The use of advanced MPI libraries, such as the Intel MPI Library, enhances network performance through multi-fabric message passing and reduces latency for inter-node data transfer [12]. Additionally, the availability of high-bandwidth technologies, such as NVIDIA NVLink and InfiniBand, facilitates the quick and low-latency transfer of data between GPUs, which is essential for parallelized deep learning jobs. All the network optimizations combine to achieve the maximum overall throughput and responsiveness of the system in time-critical applications such as intrusion detection and anomaly detection in satellite-linked IoT systems.

#### Network.

A scalable and high-performance networking system is critical in enabling efficient communication between compute nodes and GPUs in an HPC cluster. Efficiently optimized networking has a vitalimpact on the system’s capability to process large-scale, real-time applications, such as deep learning-based security monitoring in IoT networks enabled by satellites. The use of advanced MPI libraries, such as the Intel MPI Library, improves network performance through multifabric message passing and reduces latency for inter-node data transfer [12]. Additionally, the availability of high-bandwidth technologies, such as NVIDIA NVLink and InfiniBand, facilitates the quick and low-latency transfer of data between GPUs, which is essential for parallelized deep learning jobs. All the network optimizations combine to achieve the maximum overall throughput and responsiveness of the system in time-critical applications such as intrusion detection and anomaly detection in satellite-linked IoT systems.

#### Optional components.

In addition to the basic components of the architecture, optional features such as high-end interconnects (e.g., InfiniBand, NVIDIA NVLink) and hardware accelerators (e.g., high-end GPUs, Intel Xeon Phi cards) can be added to enhance the performance of an MPI-capable HPC cluster [8]. These upgrades enable faster data transfer, lower latency, and improved task execution in deep learning frameworks. In the context of our envisioned system, such pieces of hardware significantly enhance the effectiveness of core operations, such as synthetic sample production, model training, and inference, which are necessary for GAN-based intrusion detection.By incorporating these sophisticated components into an MPI-capable HPC system, deep learning models such as GANs can be utilized more efficiently to enhance security mechanisms in satellite-connected IoT systems. This incorporation addresses the significant issues associated with large-scale data processing and real-time analysis, ultimately contributing to the development of scalable, adaptive, and high-performance intrusion detection systems.

## Background and related work

Securing IoT sensors/ devices communicating to centralservers/ clouds is a challenging task and it becomes more difficult when they use satellite link as a medium. This is because of the vulnerabilities associated with satellite communication. Nevertheless, combining traditional networking protocols like Ethernet and using TCP/IP-based analysis can work towards a more secure and sustainable security design. As deduced from the literature survey and tabulated in Table 1, the deployment of the IDS-integrated proposed architecture in the central HPC cloud presents a potential line of approach towards improved security.

**Table 1:** Listing of papers in Literature Survey.

S. No	Year	Ref	Author(s)	Accuracy	Deep Learning Model	Focus
1	2022	[20]	Wang et al.	92.16	CNN	Intrusion Detection
2	2023	[21]	Sankaran et al.	98.6%	RMC-CNN	Anomaly Detection, Energy Eﬃciency
3	2023	[22]	Alabsi et al.	98.09%	Dual CNN	Feature Selection, Attack Detection
4	2023	[23]	Rahim et al.	94%	Logit-boosted CNN	Anomaly Detection, Face Recognition
5	2023	[24]	Prasath et al.	99.476	Feature optimization, IDS	DDoS attack defense
6	2019	[25]	Liu et al.	96.70%	GRU, SVDD	Anomaly detection
7	2021	[26]	Rehman et al.	99.69%	GRU	DDoS attack detection
8	2023	[27]	Zhu et al.	99.32%	Multi-neural network fusion	Multi-Feature fusion
9	2023	[28]	Bokka& Sadasivam	95.51%	GRU	RPL attack detection
10	2023	[29]	Sagar et al.	Not mentioned	CNN-GRU	Intrusion Detection
11	2022	[30]	Banaamah& Ahmad	99%	Proposed CNN, LSTM, GRU	Intrusion Detection
12	2023	[31]	Altaf et al.	97.64%	Node-Edge Graph Convolutional Network	Intrusion Detection
13	2023	[32]	Altaf et al.	98.91%	Multigraph Neural Network	Intrusion Detection
14	2023	[33]	Esmaeili et al.	Not mentioned	Graph Neural Network (GNN)	Malware Detection
15	2021	[34]	Alkahtani& Aldhyani	89.64%	CNN-LSTM	Botnet attack Detection
16	2020	[35]	Wang & Lu	98.30%	XGBoost, LSTM, Fusion model	Anomaly Detection
17	2021	[36]	Sinha et al.	99%	LSTM-CNN	Cyber threat Detection
18	2021	[37]	Azumah et al.	98.00%	LSTM	Intrusion Detection
19	2024	[38]	Kilichev et al.	96%	CNN, LSTM, GRU	Intrusion Detection
20	2024	[39]	SZ Ahmad et al.	94.6%	Autoencoder-CNN	Intrusion Detection and IoT

Our strategy leverages two models to maximize intrusion detection. Other research has investigated different machine learning methods and architectural changes to counterbalance similar issues. As an example, Prasad et al. [40] and Illiashenkoet al. [41] recommend network segmentation and isolation as a good approach. Here, the network is segmented into several zones of security based on the sensitivity and importance of devices and data being connected. Critical infrastructure can be separated from general-purpose IoT devices, thereby greatly reducing the attack surface.

Wang et al. [42] and Cao et al. [3] also suggest securing satellite communication links through the use of blockchain-based authentication and encryption methods, thus providing a secure virtual private network VPN-like connection between IoT devices and the central cloud. Satellite edge computing has also been put forward as a means of potential latency reduction and decentralization of processing tasks closer to data sources, enhancing security and performance in satellite-enabled IoT systems [42].

To address localized attacks within IoT systems, Rashid et al. [43] proposed an innovative approach by directly placing Host based intrusion detection agents on every IoT sensor or device. Their research entailed extensive experimentation with file integrity monitoring, behavioral anomaly detection, and the inspection of system-generated logs. Special emphasis was placed on logs that generated alerts against anomalous behavior, allowing for timely detection and response to possible intrusions.

Supporting this host-centric strategy, Pandey et al. [44] deployed a Network based IDS in every access point to the network (i.e., cloud ingress). This system aimed to capture and block attacks by tracking known malicious payloads, aberrant traffic signatures, and deviations from normal network activity. Their system was scalable across various types of network infrastructures, such as Ethernet-based, mobile, and satellite communications environments.

In another work, Bansla et al. [45] proposed a Hybrid Machine Learning-based Intrusion Detection system, utilizing anomaly detection methods to create a dynamic reference model for IoT network security. Their method identified deviations from a set behavioral baseline as possible indicators of an attack. Through the ongoing update of the baseline model to incorporate changing traffic patterns and emerging threat vectors, their system proved capable of adapting to new forms of cyber threats.

To remain agile in response to newly emerging cyberattacks, Pandey et al. [44] incorporated real-time threat feeds into their intrusion detection algorithms. These threat feeds were utilized to enhance detection accuracy, expand threat coverage, and optimize the performance of the detection framework. By dynamically adding external threat indicators, their system showed enhanced agility in responding to changing attack patterns.

Today’s IoT infrastructures have revolutionized the way devices interact with each other, bringing greater convenience and operational efficiency to various fields. IoT applications today range from domestic devices like smart speakers and thermostats to mission-critical industrial systems employed for predictive maintenance and environmental monitoring [13,46]. Nonetheless, the widespread expansion of IoT implementations has also created massive cybersecurity challenges, especially in satellite-enabled IoT networks where sensitive information is often communicated through unsecured channels. A study in [10] found that nearly 98% of IoT traffic was unencrypted, leaving private and sensitive information extensively vulnerable to cybersecurity threats.

Recent advancements in deep learning, particularly in the area of Generative Adversarial Networks (GANs), have opened up promising directions for enhancing IoT security. These developments have informed our solution strategy for utilizing GANs to address class imbalance issues in intrusion detection datasets and to improve detection performance. Momentum Contrastive Learning techniques have been suggested for enhancing representation learning that is crucial for effective anomaly detection and secure data exchange in IoT environments [8,11].

In addition, the implementation of self-supervised learning techniques, specifically for video and image recognition, has been found to hold promise in complementing security measures. Such techniques facilitate more efficient monitoring, enabling the detection of unauthorized access and suspicious behavior in real-time [8,12,13].

Kalpana et al. [47] developed a behavioral analysis-based intrusion detection system in their research, which involves monitoring the behavioral patterns of IoT sensors/ devices as they interact with the central cloud infrastructure. This approach targets the recognition of unusual events, such as outliers in data transmission volumes or illicit access attempts. These anomalies from expected behavior are marked as potential security incidents. Depending on the severity of the anomaly detected, countermeasures such as sending out alarms or blocking access are implemented in real-time.

In addition to this paradigm, Dalal et al. [48] leveraged the computational capabilities of cloud infrastructure to process network traffic and data generated by IoT sensors/ devices in large amounts. Their research highlighted the application of sophisticated analytical methods to identify advanced and zero-day attacks that often go unnoticed with traditional detection methods. With the use of scalable cloud-based resources, their system proved capable of processing high-throughput data streams while maintaining efficient and responsive threat identification.

IoT solutions based on satellites face several unique challenges, including high operational costs and the complexity of integrating new communication technologies, issues that can hinder their widespread adoption [4]. However, breakthrough innovations are geared towards countering these obstacles. The scaling of satellite IoT networks depends heavily on the establishment of standardized protocols and effective data management plans, especially in supporting strong asset tracking and environmental sensing in hard-to-reach or remote areas [11]. In addition, combining HPC architectures with deep learning schemes, such as our proposed DLGAN approach, offers a promising direction for enhancing data processing performance and strengthening security. Recent techniques such as knowledge distillation are also being investigated to improve image generation and anomaly detection operations in resource-limited systems [18,49].

A thorough analysis of the available literature suggests that the performance of intrusion detection models significantly improves when deep learning (DL) methods are employed. Yet, most previous works have focused on learning a limited number of attack types, which, although resulting in high accuracy, restrict the practical scope of applying such models. Even a high-level summary of the work surveyed indicates that higher threat detection accuracy in IDS is usually realized when detection is limited to a small number of known threats, even when ensemble methods of multiple DL algorithms are employed.

In response to these limitations, our study emphasizes architectural updates, the use of real-world, large-scale, and balanced datasets, and the implementation of fine-tuned, hybrid algorithms tailored to various types of attacks. In response, our study involved a thoughtful assessment of the computational resource needs of our experiment, which influenced the structure of our solution. Notably, however, a one-layered solution is insufficient for robust security. Instead, a multilayered, integrated intrusion detection system, which includes several complementary approaches in unison, is needed to detect and deter advanced threats with confidence. Continuous monitoring, periodic tuning, and dynamic adaptation of models are essential for protecting satellite-linked IoT devices that communicate with HPC clouds.

## Challenges and motivation

### Challanges

The most significant challenge we faced in our study was selecting an appropriate dataset that matched the objectives of our experiment. Through a comprehensive comparative analysis of existing IoT security datasets, as presented in Table 2, the Edge-IIoTSet (2022) dataset proved to be the most suitable for our research [66,67]. Even an initial comparison of the dataset choices revealed a greater diversity of classes, sensor coverage, and data quality in Edge-IIoTSet. The dataset comprises data from 10 IoT/IIoT sensors. It spans 15 classes, including one normal class and 14 classes of different attacks, thereby providing an extensive and balanced sampling distribution for training and testing intrusion detection models.

**Table 2:** Literature survey of the referred datasets.

S. No	Ref	Dataset	Data features and type	Size	Type
1	[50]	TON-IoT	Telemetry, normal/6 attacks	2.5 million	Real-World
2	[51]	NSL-KDD	Network data, normal/attack	125,973	Synthetic
3	[51]	UNSW-NB15	Network data, normal/attack	2.5 million	Real-World
4	[52]	IoT-23 Dataset	Network data, normal/attack	2.3 million	Real-World
5	[53]	CICIDS2017	Network data, normal/attack	2.8 million	Synthetic
6	[54]	BoT-IoT	Network data, normal/4 attacks	3.4 million	Real-World
7	[55]	IoT-BDA	IoT Botnet, Malware Analysis	4077	Real-World
8	[56]	50K-IPD	Malicious URLs	52,000	Real-World
9	[57]	DNS dataset	Malicious domain names	88,000	Real-World
10	[58]	ZeuS Tracker	ZeuS malware samples	10,000	Real-World
11	[59]	Feodo Tracker	Feodo malware samples/feeds	10,000	Real-World
12	[60]	RanSAP	7 Ransomware/5 benign samples	1495	Real-World
13	[61]	MBB-IoT	DDos attack, normal/7 attacks	2 million	Real-World
14	[62]	CICIoT2023	Network attack, normal/7 attacks	46 million	Real-World
15	[4]	Edge-IIoTSet	10 Sensors normal/15 attacks	1.3 million	Real-World

Among the competing datasets, the TON-IoT dataset, developed by Alsaedi et al. [50], presents a valuable collection of telemetry data, including both normal and malicious samples under various realistic settings. It features network traffic characteristics and subcategories of attacks designed to represent real-world IoT cyberattacks. Nevertheless, although TON-IoT presents valuable information, it lacks some attack settings and hardware diversity required for deep learning models suitable for HPC and satellite-integrated systems.

In contrast, the Edge-IIoTSet proposed by Ferrag et al. [4] was specifically developed to support the testing and development of IDS for distributed and centralized IoT/IIoT infrastructures, as well as satellite-based communication systems. Most importantly, Edge-IIoTSet includes previously underrepresented attack vectors, thereby increasing its relevance in contemporary threat scenarios. These factors made the dataset most suitable for our research goals, particularly in constructing and testing a scalable, DL-based IDS framework.

#### Challenges in IoT security.

This subsection identifies the essential challenges related to IoT device security, with special emphasis on Intrusion Detection System design and implementation in satellite-connected and HPC-integrated systems. As IoT technology continues to grow and expand into various spheres, its security challenges become increasingly crucial to address to ensure system integrity, confidentiality, and availability.

The increasing heterogeneity, constrained computational power, and distributed topology of IoT infrastructures bring in tremendous complexity to the deployment of potent IDS solutions. These complexities are then compounded in satellite communication and cloud-based HPC settings. A clear comprehension of these challenges is necessary for the formulation of adaptive and resilient security mechanisms.

**Fig 2:**
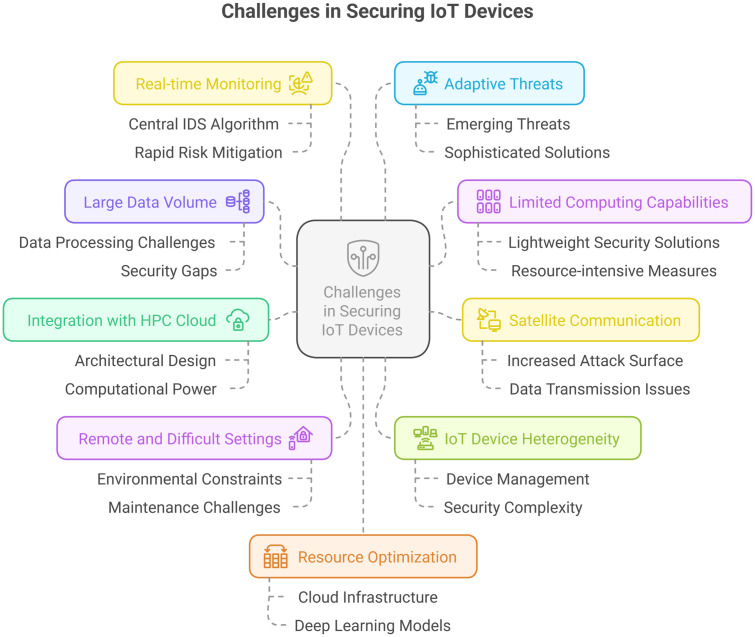
Challenges in securing IoT devices infrastructure having connectivity to HPC cloud via satellite [63].

Fig 2 is a pictorial overview of the significant security and operational issues faced in such environments. The following subsections provide a detailed elaboration of each issue, highlighting the urgent need for innovative, scalable, and resource-frugal intrusion detection frameworks that are best suited to the peculiar features of IoT environments.

#### Large data volume.

One of the primary obstacles to securing IoT infrastructures is the large amount of data generated by IoT sensors/ devices, which presents significant challenges for real-time analysis and processing. The large volume of network traffic, when combined at a central server, can overwhelm traditional IDS, hindering their ability to detect and respond to threats promptly. As noted by Rashid et al. [43], conventional IDS architectures tend to be less scalable and computationally inefficient in handling high-throughput data environments, thereby creating security gaps and vulnerabilities. This weakness highlights the need for enhanced, scalable IDS models that can support the data-intensive characteristics of IoT networks without compromising detection accuracy or system performance.

#### Limited computing capabilities.

Most IoT devices face severe resource constraints, including limited processing capacity, memory, and energy reserves. Such restrictions pose enormous challenges for implementing computationally demanding security measures. The limited computing resources on the device render conventional security methods, including standard IDS, inoperable for deployment in such scenarios. Consequently, resource-intensive IDS frameworks become unable to satisfy the operational needs of IoT systems. This requires the creation of lightweight, adaptive, and effective security solutions specifically designed for low-power and resource-limited IoT devices, without compromising detection capabilities or response.

#### Satellite communication.

IoT devices often utilize satellite communication, particularly in remote or inaccessible areas where ground-level connectivity is unavailable. This facilitates unbroken connectivity but severely increases the attack surface, making them highly vulnerable to cyberattacks, eavesdropping, and other network-based attacks [42]. Satellite transmissions typically encapsulate data in the form of Ethernet packets, which can be easily intercepted or tampered with, thereby presenting additional security issues.

Additionally, the slow processing and limited bandwidth of IoT devices further complicate these issues. In most instances, it is not possible to forward large amounts of sensor data to a centralized server for inspection because of these resource limitations. This restriction makes it challenging to implement conventional intrusion detection mechanisms and negatively impacts the real-time detection and prevention of local security threats [40]. Thus, substitute solutions, such as distributed or lightweight IDS specific to satellite-connected IoT environments, are crucial for ensuring timely and dependable threat detection.

#### Integration with HPC cloud.

Because most IoT devices require connection to a central server for management, monitoring, and analytics, integrating these devices with a centralized HPC cloud becomes crucial for ensuring both security and effective data processing. Nonetheless, such an integration is an architecturally challenging task that requires careful system design and implementation. Conventional IoT infrastructure lacks the computational capacity to handle sophisticated security measures locally; therefore, offloading computationally intensive tasks to a central HPC environment is a tactical approach.

In line with this perspective, our work leverages the processing capabilities of HPC to enhance intrusion detection and threat response capabilities [64]. By offloading computationally intensive security functions to the cloud of the HPC, we provide real-time detection and quick response to cyber threats without overwhelming the limited-resource IoT devices. This design not only facilitates scalable data processing but also enhances the security stance of satellite-connected IoT spaces through centralized cognition and distributed perception.

#### Challenging and remote environments.

IoT devices and sensors are typically situated in remote, inaccessible, or hostile environments, where maintenance and physical access are, by their nature, challenging. These environmental factors significantly complicate the process of maintaining data integrity and device security, as the devices are exposed to a range of physical and cyber threats. Limited accessdelays detection and response to potential security incidents, thereby increasing the likelihood of prolonged exposure to malicious operations. As such, situations like this call for the deployment of strong, self-sustaining, and remotely controlled security solutions capable of supporting timely anomaly detection and intervention without constant physical interaction.

#### IOT device heterogeneity.

Heterogeneity of IoT devices and sensors adds another layer of complexity to their management and security. Each type of device has a different architecture, communication protocol, and functional behavior, which gives rise to differential attack surfaces and differing vulnerabilities. This variety makes it significantly more challenging to establish uniform security frameworks and detection methods. Within our research, we overcome this hurdle by examining ten different types of IoT sensors/devices, thus encompassing the broad range of variability that can be expected in actual deployments. Such a method brings to the forefront the complexity of protecting an IoT ecosystem that is heterogeneous and emphasizes the necessity of adaptive security technologies that can be effective in multi-device environments [7].

#### Real-time monitoring.

Real-time threat detection and deterrence are essential for sustaining the security and resilience of interconnected IoT ecosystems. This capability for real-time monitoring and response to abnormalities enables the swift detection and mitigation of emerging threats, thereby reducing the impact of cyberattacks. To enable this, our system design utilizes a centralized Intrusion Detection System (IDS) algorithm on a high-performance central server, facilitating effective, timely threat detection and response. This centralized method ensures the timely generation of alerts, even in the case of massive and heterogeneous data streams from distributed IoT devices, thereby improving the overall security posture of the network [42].

#### Adaptive threats.

IoT device cyberattacks are continuously evolving, making the threat environment dynamic and significantly more challenging for conventional IDSs. These traditional systems often fail to identify new or zero-day attacks because they heavily rely on predefined rules or known attack signatures. This accelerated evolution of threat vectors necessitates the implementation of more intelligent, adaptive, and advanced security solutions that can learn and evolve in response to emerging threats. As suggested by [41], the use of sophisticated techniques, including deep learning and generative models, presents a viable direction for improving detection efficiency and ensuring resilience against ever-evolving cyberattacks.

#### Resource optimization.

Making the best use of available resources throughout the entire cloud infrastructure, including servers, workstations, and GPUs, is crucial for providing effective and scalable security in the IoT environment. Achieving a balance between protection and performance is a challenging task, particularly for heterogeneous systems that lack sufficient resources. To overcome this problem, we suggest implementing intrusion detection methods combined with deep learning algorithms on central cloud servers, along with advanced resource optimization techniques, and incorporating strong and adaptive security mechanisms, as suggested in [6].

**Fig 3:**
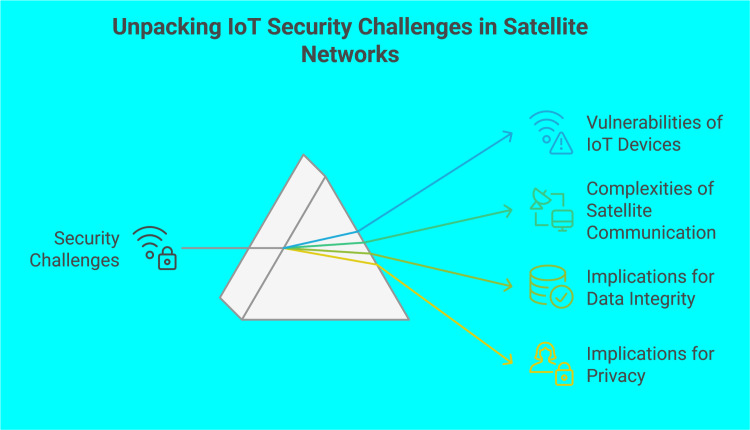
Challenges in securing IoT devices in satellite networks [63].

Finally, the above challenges, illustrated below in Fig 3, highlight the inherent intricacy of IoT infrastructures’ security, especially in cases where satellite communication and HPC integration are involved. Overcoming these challenges requires creative measures and the enhancement of sophisticated, context-sensitive security solutions specifically designed for the distinct operational and architectural features of IoT devices and sensors.

### Challenges of satellite communication

#### Latency and bandwidth constraints.

Satellite communication links inherently experience greater latency and reduced bandwidth compared to land-based networks. Such restraints pose extraordinary challenges to the deployment of security protocols reliant on real-time data processing and transmission. The escalated delay and restricted throughput can impair the timely operation of intrusion detection algorithms and hinder timely security responses, ultimately degrading the overall responsiveness and performance of the security framework in satellite-enabled IoT systems.

#### Signal interception.

Satellite communications are more vulnerable to interception compared to terrestrial channels of communication, and thus, the risk of data compromise is greater. Attackers or eavesdroppers would be able to exploit this weakness to intercept and potentially manipulate sensitive information exchanged between IoT devices and the mother HPC cloud. This vulnerability poses a serious threat to the confidentiality and integrity of the data, particularly in instances where strong encryption and authentication methods are lacking or inadequate...

#### Jamming and spoofing.

Wireless transmission of satellite signals makes them susceptible to jamming and spoofing attacks. This threat can cause interference in communication channels, potentially leading to denial-of-service (DoS) situations. These interferences can critically affect the performance of IoT devices and sensors during operation, compromise the integrity of services offered by them, and lead to failures in the timely communication and processing of data at the HPC cloud. As a result, the continuity and integrity of mission-critical applications that depend on satellite-enabled IoT infrastructure can be compromised.

### Implications for data integrity and privacy

#### Data breaches.

The intrinsic susceptibility of both satellite communication channels and IoT devices/sensors to data breaches, due to their limited resources and exposure to open environments, can potentially expose sensitive information to unauthorized parties or eavesdroppers. Such data breaches might have severe cybersecurity consequences for individuals and organizations that heavily depend on the confidentiality, integrity, and availability of data communicated for operational and decision-making purposes.

#### Loss of trust.

Unaddressed security breaches can severely undermine trust in IoT infrastructure and HPC cloud services, causing caution or reluctance in embracing these technologies. Not only do such breaches compromise operational integrity, but they also hinder stakeholders from realizing the full innovation potential of IoT and HPC-powered systems. A lack of trust can ultimately hinder technological progress and delay the realization of its benefits in key sectors.

#### Regulatory compliance.

Organizations are increasingly being asked to adhere to strict regulatory frameworks that require stringent measures for data protection and privacy. Inadequate protection of IoT devices and satellite communication channels not only puts sensitive information at risk but also can lead to non-compliance, resulting in legal issues and significant financial penalties.

As discussed in this paper, the multidimensional security threats posed by IoT devices communicating with an MPI-enabled HPC cloud through satellite communications necessitate a holistic and dynamic security strategy. Mitigation of the security threats requires augmenting the intrinsic security features of IoT devices, implementing strong IDS, and ensuring the confidentiality and integrity of satellite communications.

With the rapid uptake of IoT and HPC technology, the requirement for prioritizing security has never been more imperative. As a measure, this research introduces a new Deep Learning-based Generative Adversarial Network (DLGAN) as an IDS solution, specifically designed for IoT ecosystems integrated with HPC clouds via satellite communication. The architecture transfers computation-intensive operations to a centralized HPC infrastructure, leveraging its high processing capacities to identify threats more effectively. In addition, DLGAN addresses the issue of imbalanced and limited data samples by generating synthetic attack data, thereby enhancing the accuracy of detection and system robustness against advanced cyberattacks.

## Motivation

The primary objective of this study is to improve the security of communication for IoT devices, particularly in satellite-enabled HPC scenarios. Several key motivating factors that shape our proposed solution and guide the direction of this research include several pivotal points. Not only do these motivating factors justify the need for our work, but they also highlight the contributions and key findings presented in this research paper.

### Ever-increasing adoption of IoT and cloud computing.

En masse deployment and adoption of IoT devices have increased in tandem with the growing application of cloud computing technologies. While IoT devices are increasingly used as data generators, and cloud platforms serve as the primary venues for data aggregation and analysis, the severity of security concerns associated with them has risen dramatically. The increasing use of connected systems and centralized cloud-based infrastructure for data processing expands the attack surface, making the necessity for strong, scalable, and dynamic security solutions more critical than ever.

### IoT device security problems.

Since they are extensively deployed in various domains and inherently limited in their security functionalities, IoT devices are highly susceptible to a wide range of cyberattacks, including Man-in-the-Middle (MITM) and Denial-of-Service (DoS) attacks. Most of these devices act as cameras, gathering sensitive environmental and operational data while executing mission-critical functions. This makes them highly vulnerable to attacks by malicious actors, as noted in [65]. Even if single data points are not suspicious, attackers can deduce sensitive information by aggregating and correlating sensor outputs, thereby breaching data confidentiality and system integrity.

### Satellite connectivity.

Satellite-based connectivity is a vital, and often the sole feasible option, for numerous IoT deployments, especially in geographically remote, inaccessible, or isolated areas. Although it facilitates wide and unbroken coverage, it also poses significant cybersecurity threats, such as eavesdropping, Man-in-the-Middle (MITM) attacks, Denial-of-Service (DoS) attacks, and other advanced threat vectors [65]. In this research, we confront these issues through the investigation of 14 different forms of cyberattacks to construct a robust and adaptive intrusion detection system specific to satellite-facilitated IoT environments.

### An integrated IDS technique is suggested.

Security for IoT involves multiple layered challenges that must be addressed in context. Whereas conventional IDSs are often advised against to counteract these attacks, they generally struggle to operate in the specific conditions of IoT networks. IoT devices are particularly vulnerable due to their limited computational power [6] and the continuous generation of substantial amounts of data [45], which is typically collected and analyzed at a central cloud platform. To address these drawbacks, this research proposes a Deep Learning-based IDS model, trained explicitly on IoT-related datasets, and implemented at the core server level, leveraging the computational power of HPC to enable scalable, real-time, and precise threat detection.

### Need for real-time monitoring.

To effectively identify and counter security threats, an IDS must operate in real-time, enabling the instant identification and blocking of anomalies. This ability is vital in mission-critical environments, where even slight delays in detection can lead to considerable operational or security compromise [45]. Within the architecture, we propose and demonstrate the viability of incorporating IoT devices with HPC clouds through satellite communication links, thereby enabling centralized real-time monitoring and sophisticated threat detection, while ensuring communication efficiency and system responsiveness.

### Emerging threat landscape.

Cyberattacks are constantly increasing in terms of complexity and sophistication, presenting serious threats to IoT devices, both from known vulnerabilities and unknown or zero-day attacks. The ever-changing threat landscape necessitates the development of sophisticated, adaptive, and resilient security solutions that can respond to evolving challenges in real-time [41].

### Resource optimization.

Effective use of the minimal computational resources deployed on IoT devices, without overloading their processors, while ensuring strong security, is a crucial and ongoing challenge in designing secure IoT systems [43].

### Integration with HPC cloud.

By integrating IoT devices with a central HPC cloud, one can avail higher processing capabilities for data analysis, threat identification, and response. However, this integration must be executed safely to protect sensitive IoT data from potential cyber threats [64]. Securely maintaining the integrity of IoT devices connected to central HPC systems is crucial in preserving the overall system’s resilience and defense against intrusions. This is especially crucial in the context of providing constant availability of IoT services in mission-critical applications, where security violations can result in severe operational impacts [40].

### Practical application.

This research closes the gap between the security issues of IoT deployments integrated with satellite-connected HPC cloud infrastructure and the practical, scalable solution development process, guided by a thorough literature review. It brings to the forefront the necessity for a new intrusion detection methodology directly addressing the specific constraints and weaknesses of such an environment.

In summary, the primary motivation for this study is to counter the escalating cybersecurity threats that increasingly target IoT devices in satellite-enabled HPC cloud environments, where traditional security measures may be insufficient. With this purpose, the research proposes a Deep Learning-based Generative Adversarial Network (DLGAN) as a novel IDS solution, which is expected to improve security, decrease false positives, and improve the resilience of IoT infrastructures against known and unknown cyber threats.

## Proposed architecture

**Fig 4:**
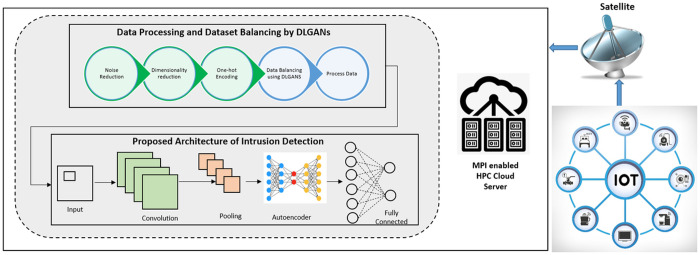
Proposed architecture.

In this paper, we introduce a new architecture, DLGAN, that aims to enhance intrusion detection and cyber threat resilience by leveraging the processing capabilities of high-performance cloud infrastructure. The architecture is described in Fig 4, which gives a conceptual map of the system’s elements and their relationships. The suggested framework combines state-of-the-art deep learning models, such as Convolutional Neural Networks (CNNs), Autoencoders, and Generative Adversarial Networks (GANs), to build an adaptive and resilient intrusion detection system.

This design is custom-made to counter the given set of security threats posed by IoT devices connected to HPC clouds via satellite communication links. Leveraging the Edge-IIoTSet dataset, the system successfully detects and classifies 14 different categories of cyberattacks with impressive precision. The core elements and working flow of the proposed DLGAN design are illustrated in Fig 4, providing a clear picture of the data processing pipeline, model training, and threat classification scheme.

### Sensor layer.

This layer is comprised of IoT devices and sensors that collect data from the physical world. The information collected by these devices, either as continuous streams or as discrete blocks, is passed on to the next layer. Such communication allows for centralized observation and control by passing on the information to HPC-supported cloud infrastructure, enabling real-time analytics and decision-making support for business and industrial applications...

### Preprocessing layer.

This layer is tasked with processing the preprocessed data received from the previous layer. The preprocessing pipeline comprises critical processes like filtering, aggregation, normalization, and deletion of missing or NaN values. These processes are essential in improving the quality, consistency, and usability of the dataset, thus making it ready for successful model training. According to standard data preparation, the dataset is normalized and cleaned so that all features are scaled to a uniform range, allowing for precise analysis and practical learning. Additional steps are also taken to filter out noise and improve the signal-to-noise ratio for future processing. The preprocessed and cleaned data is then passed to the next layer for training and testing the model.

### DLGAN layer.

Deep Learning-based Generative Adversarial Networks (DLGANs) are a cutting-edge variation of classical Generative Adversarial Networks (GANs), with extensive applicability in many areas, including the security strengthening of satellite-enabled IoT networks. DLGANs utilize the basic building blocks of GAN design, i.e., a Generator and a Discriminator, both of which are realized using convolutional neural networks (CNNs). These building blocks undergo an adversarial training process, where the Generator attempts to create realistic synthetic samples of data, and the Discriminator tries to distinguish between real and generated data. This antagonistic interaction enables the model to continually enhance its data generation capacity, thereby improving overall model performance in tasks such as synthetic sample generation, data augmentation, and intrusion detection for applications involving imbalanced IoT security datasets.

### DLGAN architecture.

The design of a DLGAN builds upon the traditional GAN architecture by incorporating state-of-the-art deep learning methods, enabling it to effectively handle large datasets, such as those generated in satellite-based IoT networking environments. Within this architecture, the Generator is responsible for generating synthetic samples of data that closely match the statistical and structural properties of real-world IoT data, specifically those underrepresented samples in the dataset. These properties encompass sensor-generating readings, network traffic flow patterns, and other contextual properties essential for training an efficient intrusion detection model [9,14].

On the other hand, the Discriminator functions as a binary classifier, separating generated samples from real ones. Through the integration of deep learning architectures, especially Convolutional Neural Networks (CNNs), within the Discriminator, DLGANs can achieve substantial improvements in classification accuracy. Such improvement enables the model to more accurately detect malicious or aberrant behaviors within the IoT network, thereby enhancing the overall security posture of the system [9].

### Training process.

Training a DLGAN is a two-step, multi-iterative adversarial process based on classical GAN frameworks, further enhanced using optimization techniques specific to satellite-based IoT network security scenarios. The Discriminator and Generator are trained simultaneously, where the Generator iteratively learns to generate samples of data that closely resemble actual IoT data, to deceive the Discriminator. At the same time, the Discriminator is constantly improved to correctly differentiate between real and generated data samples [14].

To enable effective and stable convergence, sophisticated training methods such as mini-batch gradient descent are utilized. These optimization techniques enable more robust updates to the network weights, which is especially critical in the satellite-IoT scenario, where data streams are highly variable and complex [9]. The adversarial interaction between the Generator and Discriminator leads to reciprocal improvement, wherein each network learns progressively from the other’s performance. This interactive training mechanism yields a more robust and precise model, which can effectively identify cybersecurity threats in heterogeneous and resource-constrained IoT environments...

### Applications and implications.

The use of DLGANs in satellite IoT networks provides significant benefits. By producing highly realistic synthetic data, DLGANs can be utilized for various cybersecurity tasks, including anomaly detection, threat modeling, and data augmentation, all of which contribute to enhancing the overall security posture of IoT infrastructures [9,14]. These artificial data samples not only compensate for skewed or small datasets but also strengthen diversity in training and testing corpora, thereby increasing the robustness and generalization capabilities of machine learning classifiers against real-world cyberattacks.

In addition, DLGANs can play a critical role in mimicking attack scenarios, allowing security teams to test, analyze, and refine their intrusion detection and response systems ahead of time in a secure environment, without compromising the integrity of production networks. As GAN architecture becomes more refined, its integration into satellite-based IoT security platforms will push the frontiers of development of effective, dynamic solutions that can counter the burgeoning, ever-evolving environment of IoT cyber threats.

### Intrusion detection layer.

The responsibility of identifying 14 various kinds of attacks/ intrusions is delegated to this layer. Using the classification algorithms, malicious and benign samples are determined first and then assigned to one of 14 categories of attack types. If the intrusion detection layer identifies spurious activity, alerts or notifications will be initiated to system/ network security administrators on management dashboards.

### High performance computing cloud layer.

This use of this layer not only enhances the computational edge of our architecture but also is tasked with storing and analyzing the summarized data from the sensor layer. The summarized data is then passed on for analysis by a mixture of AI algorithms (CNN and Autoencoders) to identify attacks/ intrusions. The local/ nearest cloud server aggregates data originating from IoT devices/ sensors to determine intrusion. Still, before that, the modified/ balanced Edge-IIot dataset from DLGANs is employed to train the model, with the support of HPC cloud servers, which act as extended computing resources to enhance the central cloud’s ability to train a central model. The cloud enhances our architecture to handle the large amounts of data generated by IoT sensors/ devices by adding processing capacity to the central server. The AI models are trained for attack/ intrusion detection, finally. The central HPC server monitors the overall functioning of the system and triggers alarms if any attack is identified.

## Implementation and methodology

The experimentation strategy of our experiment includes a bi-phased approach. Phase one involves analyzing the imbalances in the dataset and enhancing it using our proposed method with DLGANs. Phase two entails training and testing the model using a test/train split dataset. In these phases, we encountered various uphill limitations and challenges.

The primary challenge was to select a suitable dataset that not only fulfills our aim of attack detection but also contains samples of specialized IoT devices and sensors. In our suggested architecture, IoT devices/ sensors send their data to their corresponding HPC clouds, using TCP/IP as the protocol, to achieve the objective of central management and monitoring services offered to their customers by utilizing the clouds. However, this TCP/IP-based communication is vulnerable to MITM, eavesdropping, and DoS attacks from malicious attackers. Hence, we needed to shortlist/ acquire a dataset containing a variety of different forms of attack samples and data from various real-world sensors. Fortunately, we shortlisted such a dataset reported in [4]. Accurate identification of benign as well as 14 kinds of malware is an utmost challenge. We achieved a correctness of 95.6%. It is implemented using MPI4Py and PyTorch in Anaconda Spyder 5.3.3.

This paper discusses the integration potential of Message Passing Interface (MPI) and its Python wrapper library, MPI4Py, to exploit the power of central HPC servers to enable IoT. We can use these technologies to create synthetic samples by parallelizing the work of Generative Adversarial Networks (GANs) on different nodes of an HPC cluster. This will not only enable efficient use of computational resources but also expedite overall processes.

### Introduction to MPI and MPI4Py.

Message Passing Interface (MPI) is a popular and portable system for message-passing parallel programming, designed to support process communication in parallel computing environments. MPI is widely used in HPC applications due to its high efficiency and scalability. MPI4Py is a Python interface for MPI, enabling Python developers to easily leverage the power of cluster computing resources using the MPI API in their applications.

### Imbalances in the edge-IIoTset dataset are addressed using GANs.

**Fig 5:**
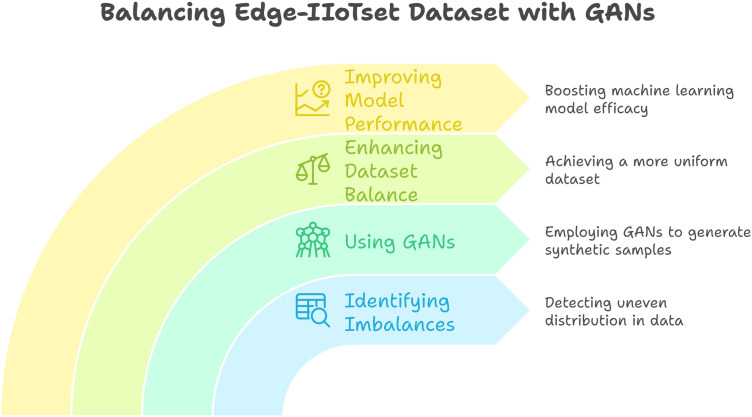
Steps involved in balancing the dataset by creating samples using GANs [63].

In data science and machine learning, imbalanced datasets are prevalent; posing a significant challenge, especially for the Edge-IIoT dataset.This paper discusses the detection of imbalances in this dataset. It suggestsusing GANs as an exploratory measure for creating synthetic samples, as depicted in Fig 5, rather than employing conventional oversampling strategies through incorporated Python APIs. Through the utilization of the strength of GANs, we will improve the balance of the dataset and consequently optimize the performance of machine learning models trained from it.

### Identifying imbalances in the edge-IIoTset dataset.

**Fig 6:**
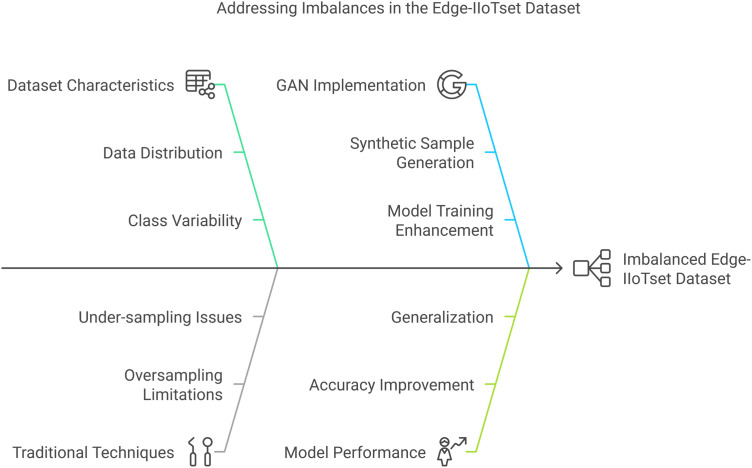
Issues regarding balancing the dataset by using GANs[63].

The initial and primary measure for resolving imbalances is to perform a proper examination of the Edge-IIoTset dataset, and then identify the Issues regarding balancing the dataset using GANs, as explained in Fig 6. The identification of imbalance entails:

### Data exploration.

Examining the malicious and benign sample class distribution within the dataset to determine whether there are any notable discrepancies or imbalances. This is done by creating visualizations in the form of histograms or bar charts that show the frequency of each class.

### Statistical analysis.

Computing measures like the class distribution ratio, which is used to estimate the degree of imbalance. A typical threshold is a ratio of 1:3 or higher, indicating a potential imbalance that may impact the model’s performance.

### Impact assessment.

To understand how these imbalances impact the model’s training and evaluation metrics, such as accuracy, precision, recall, and F1 Score. This evaluation will help us determine the best process to address the imbalance in the dataset.

### Generating samples using GANs

After imbalances are identified, the next step is to create synthetic samples using GANs. The steps are as follows:

#### Understanding GANs.

GANs consist of two CNNs, the generator and the discriminator, both of which function as rivals playing opposite roles to develop realistic synthetic data. The generator creates new samples, while the discriminator checks their authenticity.

#### Data preparation.

Preprocess the Edge-IIoTset dataset so that it is ready for training the GAN. This involves normalizing the data, dividing it into training and validation sets, removing NaN values, and one-hot encoding.

#### Training the GAN.

Coding the GAN architecture in Python and training it on the minority classes of the Edge-IIoTset dataset. The aim is to equip the generator with the ability to generate samples that closely match the minority class instances.

#### Evaluating generated samples.

Upon training, it is crucial to check the quality of generated samples. This can be done using parameters/ metrics like Inception Score or Fréchet Inception Distance (FID) to make the synthetic data similar to the real data and beneficial for training the model.

#### Integrating synthetic samples.

Lastly, in this step, the synthetically created samples are added to the original dataset, essentially gaining a balance in the class distribution. This new augmented dataset can subsequently be utilized for training machine learning models, which may result in improved performance.

#### Conclusion.

Finally, data imbalances in the Edge-IIoTset dataset must be addressed to construct reliable machine learning models. Through a determination of the imbalances and utilization of GANs as synthetic sample generation methods for dataset balancing, we can advance the efficacy of the dataset as well as enhance the overall performance of models. Not only does this eliminate the drawbacks of traditional oversampling methods, but it also taps into the capability of advanced generative models to generate high-quality synthetic data.

#### The role of GANs in sample generation.

GANs are part of a well-known group of machine learning architectures, in which a pair of sets of convolutional neural networks, a Discriminator and a Generator, operate adversarially against one another. In our case, the generator generates artificial data samples, whereas the discriminator checks their realism. This competitive adversarial process enables GANs to generate high-quality synthetic data samples that can be utilized in various applications, such as network security, IoT network attack detection, and satellite communication.

#### Distributed sample generation with MPI and MPI4Py.

**Fig 7:**
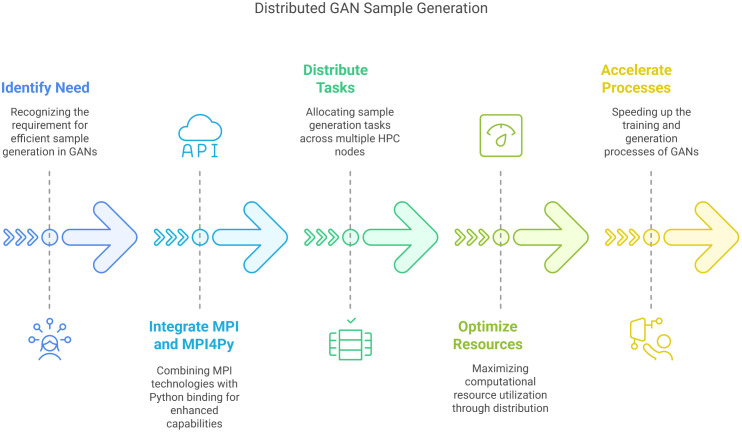
Generation of samples by GANs over a distributed infrastructure [63].

For efficiently distributing the task of sample generation by GANs on an HPC cluster, wecanutilizeMPI4Py, which is built on top of MPI as depicted in Fig 7. The steps below providetheentireprocess:

#### Initialization.

Establish the MPI environment as well as MPI4Py. All nodes of the HPC cluster will initialize their respective MPI instances with shared digital signature keys and shared SSH keys, allowing for smooth SSH access to all nodes to submit jobs and retrieve their executed output.

#### Data partitioning.

Distribute and split the input data across the available nodes. Each node will be tasked with creating a subset of samples based on the GAN model.

#### Sample generation.

Each node runs the GAN model on the dataset assigned to it and produces samples independently. Reducing the overall computation time by a large margin through parallel execution.

#### Communication.

Use Message Passing Interface to facilitate communication between nodes of the cluster. Nodes can share generated samples and SSH keys as needed, ensuring that the overall architecture remains synchronized and well-communicated seamlessly.

#### Aggregation.

Once all the nodes have finished generating samples, results can be summed up on the master nodes through secure shell communication of MPI. This may include gathering all generated samples into a common repository at the master node for further processing.

#### Finalization.

Upon aggregation of all samples from all the nodes, the MPI environment can be closed, and the produced samples can be used for subsequent tasks for further model training.

### Empirical benefits of using MPI4Py in IoT HPC servers

#### Scalability.

The ability to scale up the sample generation process over multiple nodes opens the way for dealing with larger datasets to train more sophisticated models.

#### Efficiency.

Distribution of the workload over several nodes decreases the time taken for sample generation, thus allowing for quick iterations in the training of models.

#### Resource optimization.

Proper utilization of the entire capacity of the HPC cluster guarantees optimal use of computational resources and effective allocation of resources.

**Fig 8:**
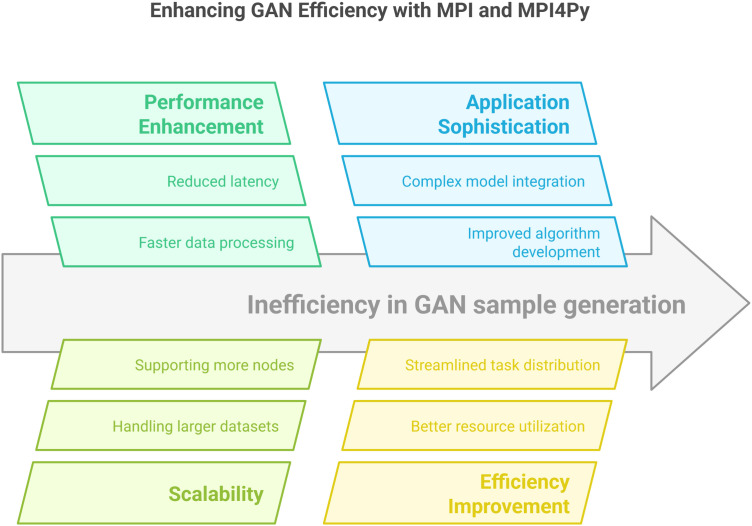
Enhancement of the efficiency of the generation of samples by GANs over an MPI/MPI4Py-enabled distributed infrastructure [63].

Lastly, the Fig 8 explains that thecombinationof MPI and MPI4Py, along with the core of IoT HPC servers, provides a robust solution for distributing the synthetic sample generation process by GANs across multiplenodes. This solution not only enhances performance but alsoopensthedoorto more advanced solutions in the field of AI/ML-basedsynthetic data generation.Utilizingtheabovetechnologies, researchers and developerscan enhance the efficiency and scalability of their security solutions,aswell astheir implementations.

## Experimental setup

### Preprocessingsetup of the dataset.

A publicly available Cybersecurity dataset, named “Edge-IIoTset,” containing benign and malicious traffic of IoT &IIoT, is downloaded from Kaggle [50]. The count of dataset splits is provided in Table 3. The dataset consists of 1909671 samples. The dataset consists of two classes: Malicious and Benign. The malicious class is also divided into 14 subclasses, each with a different type of attack. The sample size for each subclass is provided in Table 3.

**Table 3:** Details of edge-Iot-Iiot dataset samples.

Class	Comments	Count
“Normal”	No Upsampling-Required	1363998
”DDoS-UDP”	No Upsampling-Required	121567
”DDoS-ICMP”	No Upsampling-Required	67939
”SQL-injection”	No Upsampling-Required	50826
”DDoS-TCP”	No Upsampling-Required	50062
”Vulnerability-scanner”	No Upsampling-Required	50026
”Password”	No Upsampling-Required	49933
”DDoS-HTTP”	No Upsampling-Required	48544
”Uploading”	No Upsampling-Required	36807
”Backdoor”	No Upsampling-Required	24026
”Port Scanning”	No Upsampling-Required	19977
”XSS”	Upsampling-Required	15066
”Ransomware”	Upsampling-Required	9689
”Fingerprinting”	Upsampling-Required	853
”MITM”	Upsampling-Required	358

This paper presents an overview of the “Edge-IIoTset”. The dataset is vital for research and development in cybersecurity, especially in the context of IoT and Industrial Internet of Things (IIoT) settings. It contains a total of 1,909,671 samples, divided into benign and malicious traffic, with the malicious class further categorized into 14 specific types of attacks. Information on the split count of the dataset is presented in Table 3.

The data is organized into two significant classes: Benign and Malicious. The Malicious class is further divided into various attack types, each corresponding to distinct types of cybersecurity attacks. The following is the clear division of the dataset split count:

This dataset can serve as a valuable tool for researchers and practitioners seeking to enhance cybersecurity in IoT and IIoT systems, providing a comprehensive overview of both normal and malicious traffic flows.

### Experimental settings

#### Experimental settings of GANS.

**Fig 9:**
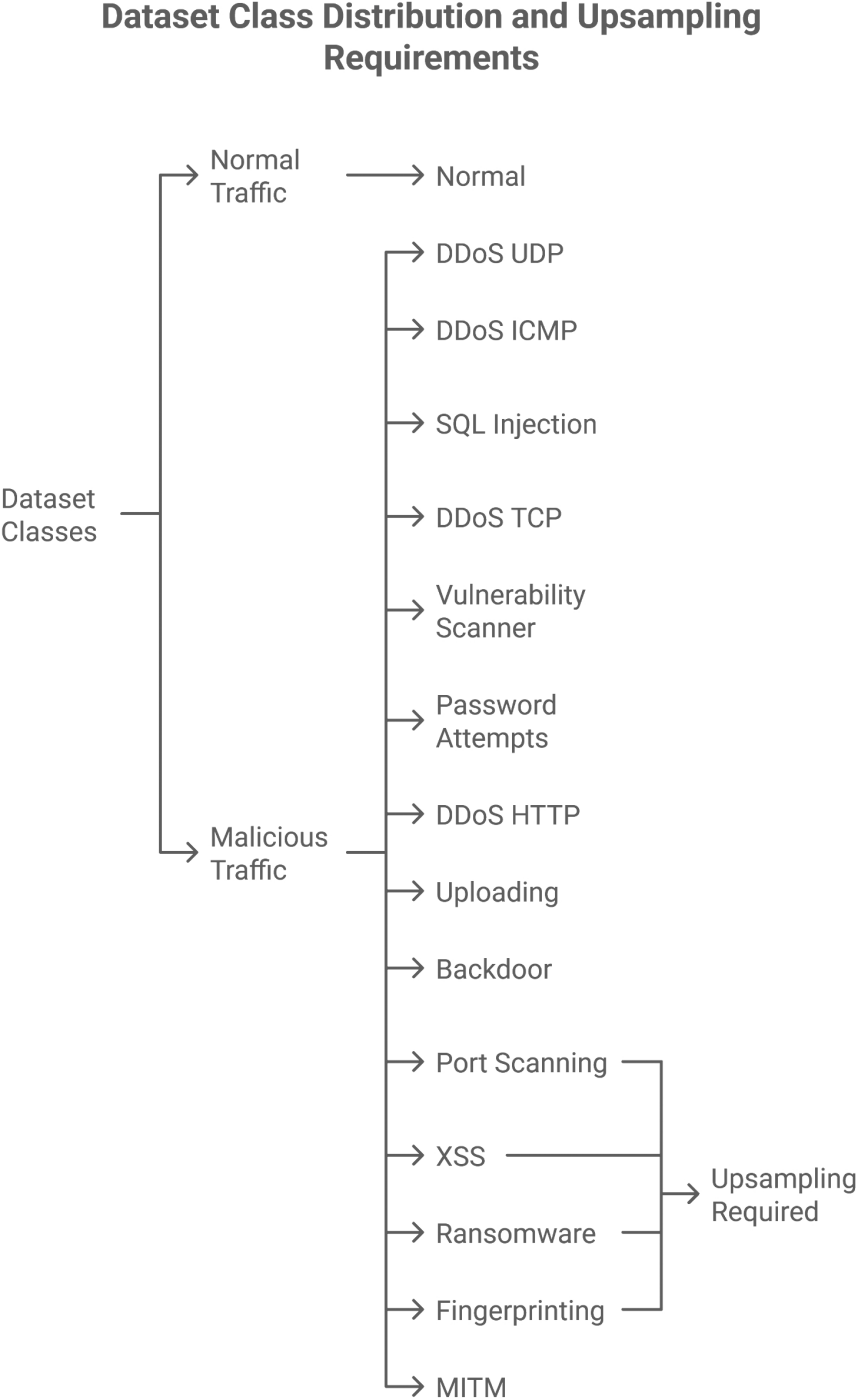
Distribution of dataset samples and their types [63].

Since the dataset is imbalanced, the columns, i.e., ‘Port Scanning’, ‘XSS’, ‘Ransomware’, ‘Fingerprinting’, and ‘MITM’, are recognized as minority classes. For this very reason, we carried out up-sampling as explained in Fig 9 above. To enhance the samples of these minority classes, we upsampleeach of the abovestated columns to 50,000, thereby increasing their contribution to our model’s decision making process. We suggest that Upsampling should be carried out using GANs.

We now present an in-depth explanation of an implementation of GANs using TensorFlow and Keras to generate synthetic samples for the minority classes in our dataset. The step we have implemented involves data preprocessing, GAN architecture specification, model training, and synthetic data sample generation. Subsequently, an explanation is provided, along with a discussion on hyperparameter optimization.

Detailed information is provided on the number of samples for each type of attack, and in the comments section, it is specified where up-sampling is required.

### Upsampling using GANs: process explanation

#### Library imports.

We start by importing the required libraries, such as NumPy, Pandas, TensorFlow, and Keras. We mount Google Drive toaccess datasets saved there, as we utilize the Google Colab platform for experimentation.

#### Data loading.

We loaded the dataset from a given CSV file of our dataset. The label column is specified, and the dataset is filtered to include only the minority classes defined in minority_classes, a list drawn from the table above.

#### Data Preprocessing.

We split the feature (X) and label (y), and the features are scaled with Robust-Scaler to reduce the impact of outliers. We scaled the features using Robust-Scaler. This scaler is less affected by outliers compared to the StandardScaler.

#### GAN Generator.

**Fig 10:**
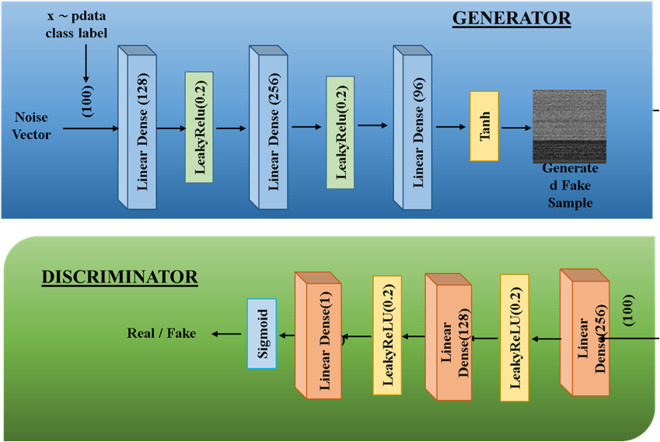
DLGAN architecture.

The generator model is specified to accept a random noise vector z, drawn from a prior distribution p_z (z), as input and produce synthetic samples G(z), aiming to mimic the real data distribution pdata(x). It has some dense layers with Batch Normalization (increases training speed and stability) and Leaky-ReLU activation functions (assists with training stability). The architecture of the generator is explained in Fig 10 below.

#### GAN discriminator.

The discriminator model takes real data x∼pdata(x) and fake data G(z) as input to examine and classify samples as real or synthetic. It is also made up of dense layers with Leaky-ReLU activations. The output following a sigmoid activation is a scalar D(x) ∈ [0,1], which is the likelihood that the input is real. Its goal is to distinguish real data from artificially produced data by G correctly. The architecture of the discriminator is explained in Fig 10 below.

#### Mathematical formulation.

We have used Class-wise Vanilla GAN (Fully Connected GAN for Minority Class Augmentation). GANs are learned using a min-max game, wherein the Generator tries to “trick” the Discriminator, and the Discriminator tries to label real vs. generated samples accurately. The value function V(G,D) is given as formula-1:


$$min_{G}max_{D}V(G,D)=E_{x\sim p_{data(x)}}\;\;\;\;\left[logD(x)\right]+{\;E\;\;\;}_{\;z\sim p_{z(z)}\;\;\;\;\;}\left[log\left(\text{1}-D\left(G(z)\right)\right)\right]$$ (1)

Here, the first term denotes the expected log-probability that the Discriminator correctly identifies real data, and the second term represents the expected log-probability that the Discriminator identifies fake data as fake.

#### Training function.

The train_gan function is responsible for training both the generator and the discriminator. It compiles both models with the Adam optimizer and binary cross-entropy loss. The training cycle involves generating fake samples, training the discriminator with both real and fake samples, and then training the generator. The Discriminator maximizes V(G,D) by enhancing its ability to distinguish real data (D(x)→1) from fake data (D(G(z))→0). This is achieved via gradient ascent on ∇θDV(G,D), where θD are the Discriminator’s parameters. The Generator minimizes V(G,D) by getting G(z) to look like real data (D(G(z))→1). Typically, this involves gradient descent on ∇θGEz∼pz(z)[log(1 − D(G(z)))], where θG are the Generator’s parameters. However, in practice, a non-saturating loss, maxGEz∼pz(z)[log D(G(z))], is used to give stronger gradients early during training.

#### Main loop.

**Fig 11:**
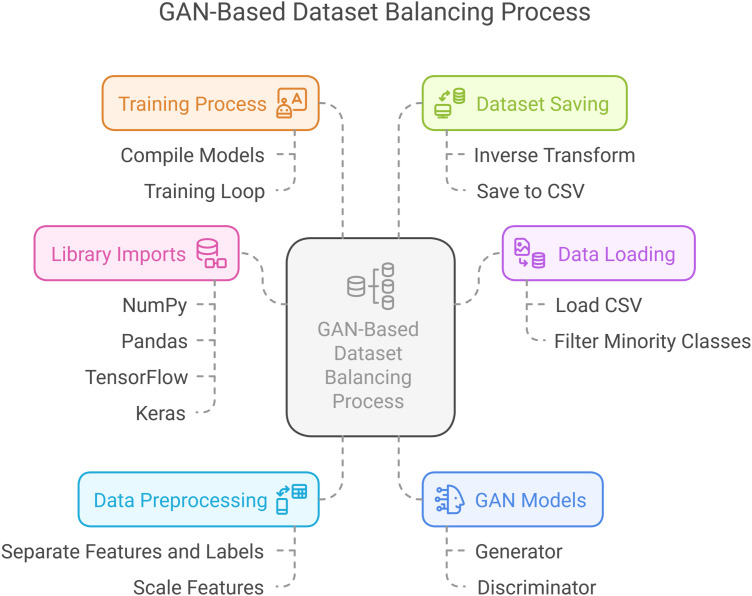
Upsampling using GANs process explanation [63].

The primary loop recapitulates over every minority class, creating a predetermined number of synthetic samples foreveryclass, which is 50000 in our case. The created samples are inverse transformed to their original scale and concatenated with the original dataset to create a balanced dataset. The whole upsampling process is depicted in Fig 11 below.

#### Saving the balanced dataset.

Lastly, the balanced dataset is saved to a CSV file. The final balanced dataset is published and made available at [66].

### Hyperparameter tuning

Our code for experimentation utilized several hyperparameters, which were tuned to enhance the performance of the GAN and improve the quality of the synthetic data generated. Below is a description of the most critical hyperparameters and an explanation of how we tuned them:

### Generator and discriminator tuning

Number of Layers: Changing the number of layerscan impact the capacity of the model. Thenumberoflayersandunitscan be modified.Increasing or adding more layers or units mightenhancethe model’s capacity, butalso increase therisk ofoverfitting.

Number of Neurons per Layer: Like the number of layers and neurons affects model capacity. Try different numbers.

Activation Functions:LeakyReLU is applied here. We tried experimenting with other activations such as ReLU or ELU, but we found LeakyReLU as a suitable option for GANs.

Batch Normalization: It is mainly good to apply it. We tested and without putting it. Testing the application of Batch Normalization layers was done by adding or removing them to observe whether it impacted training stability for the better.

By configuring these hyperparameters in an orderly fashion and observing the performance of the GAN, one can fine-tune the model for improved synthetic data generation and overall performance.

#### Trainingparameters.

*Learning Rate:* The learning rate of the Adam optimizer is highly sensitive. If the Adam optimizer value is too large, the model will not converge, but on the other hand, too lowwill mean a very slow time to train. Play around with values such as 0.0001, 0.0002 (the one that has been used in the code), 0.001, etc. The learning rate of the Adam optimizer is 0.0002. This value was adjusted by attempting smaller or bigger values and noting their impact on convergence.Wediscovered0.0002asbest.

*Beta_1:* The beta1 parameter in Adam adjusts the rate of exponential decay of the moving average of the gradient.Itwasadjustedto 0.5.

*Batch Size:* Training stability and speed depend on batch size.Increasing the batch size sometimes yields improved results, but it also consumes more memory. Powers of 2 (e.g., 64, 128, 256) were used for experiments. We adaptively vary batch size when the class data is limited. The batch size was initially 128, but was adapted depending on the available data. Experienced various batch sizes, but it affected the speed and stability of the training.

*Number of Epochs:* The number of epochs in training controls how many times the model trains. Not enough epochs, and the model will not learn properly. After a sufficient number of epochs, it will overfit. Track the losses and terminate training when they reach a plateau. The number of epochs is configured to 10 for the training function. More epochs can result in more effective training, but can potentially increase the likelihood of overfitting.

*Noise Dimension (input_dim):* The Dimension of the input noise vector to the generator is described in this variable. This can affect the diversity of output samples. On trying various values, it is recommended to set it to 100.

*Loss Function:* Binary cross-entropy is normal; we have tried Wasserstein loss and other GAN-specific losses, but we faced training instability. GANs are implemented with the following loss functions.

*Discriminator Loss* formula-2


$$L_{G}=\;-\frac{\text{1}}{m}\sum {\mathrm{\backslash nolimits}}_{i=\text{1}}^{m}[logD\left(x_{i}\right)+log\left(\text{1}-D\left(G\left(z_{i}\right)\right)\right)]$$ (2)

*Generator Loss* (Non-Saturating) formula-3


$$L_{D}=\;-\frac{\text{1}}{m}\sum {\mathrm{\backslash nolimits}}_{i=\text{1}}^{m}[\text{log}\left(D\left(G\left(z_{i}\right)\right)\right)]$$ (3)

Here, m denotes the batch size. The networks are typically deep neural networks (e.g., convolutional neural networks for our data) and are optimized using algorithms like Adam.

### Experimental settings of proposed IDs architecture

**Fig 12:**
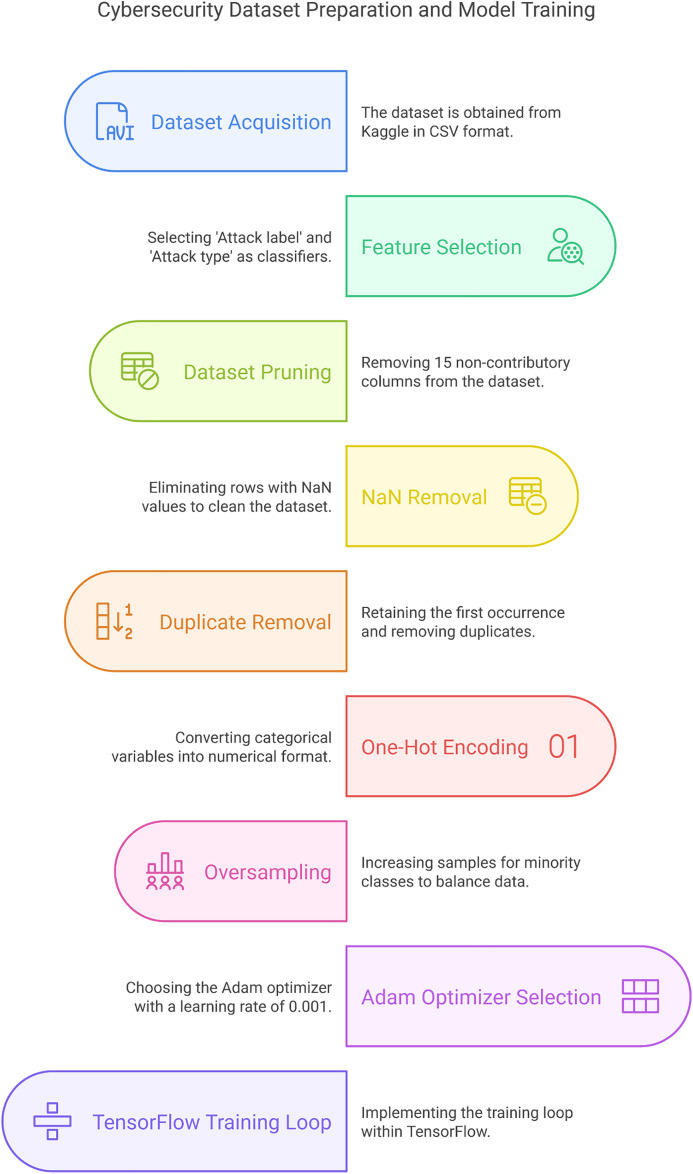
Dataset preparation and pre-processing Explanation [63].

In this section, we outline the steps taken to prepare the dataset for detailed analysis and training of a machine learning model, which aims to detect attacks in the field of cybersecurity as described in Fig 12 below. The dataset, acquired in CSV format from Kaggle [67], contains several features that were technically pruned and processed to enhance the model’s performance. The main techniques we employed were One-Hot Encoding, oversampling, and utilizing the best optimizer, such as the Adam optimizer, to ensure effective learning and preprocessing of our dataset.

#### Hardware and processing resources.

Considering the processing-intensive nature of GANs, we opted to use Google Colab to process the dataset and prepare a balanced dataset. The balanced dataset is then placed on our HP Workstationmachine, which is equipped with an Intel Core i7-10610 Processor, 512 GB NVME SSD, and 16 GB of RAM with an NVIDIA Quadro P520 GPU, to process our CNN-based IDS model.

#### Dataset acquisition and initial processing.

Initially, the dataset was acquired from Kaggle in CSV format. It served as the initial point for our dataset analysis. The dataset comprises 63 columns, out of which we identified ‘Attack label’ and ‘Attack type’ as the two key classifiersindispensable for our analysis. Subsequently,the dataset pruning was carried out by removing 15 columns. The columns were non-contributory in the model training process. The names of those columns, along with reasons, are mentioned below.

#### frame.time.

Reason: This column depicts the timestamp of when the packet was captured. On the other hand, timestamps are necessary to determine the exact time of an event’s occurrence. Still, they have no direct relevance to the detection of malicious behavior unless analyzed in conjunction with higher-level temporal features [68].

Impact: Without preprocessing (e.g., extracting time-based features like intervals or periodicity), raw timestamps do not provide detailed insights [68].

#### ip.src host.

Reason: In this dataset, this column contains the source IP address of the packet. The source IPs can indicate the origin of traffic; they are highly variable and unique in most datasets. This variability makes them less generalizable for training a model, as the model may overfit to specific test/trial LAN IPs instead of learning broader patterns [69].

Impact: Instead of random public raw IPs, derived features such as IP reputation or frequency of connections from an IP might be more useful [69]; however, in this scenario, it is not possible due to LAN IPs.

#### ip.dst host.

Reason: The reasoning is similar to ip.src host as this column comprises the destination IP address. Like source IPs, destination IPs are also highly variable and unique, making them less useful for generalization [69].

Impact: Derived features such as destination port popularity or connection patterns could be more useful/ informative [69] for training a model.

#### arp.src.proto ipv4.

Reason: Here in this dataset, the column reflects the source IPv4 address in ARP (Address Resolution Protocol) packets. ARP-related fields are protocol-specific and may not generalize well across different types of network protocol traffic. Additionally, ARP traffic is often found to be benign, and this kind of traffic is not directly indicative of intrusion attempts [70].

Impact: Unless the IDS is focused explicitly on ARP-based attacks (e.g., ARP spoofing), and model training is tailored around ARP attacks, this field is unlikely to contribute effectively [70].

#### arp.dst.proto ipv4.

Reason: Similar to arp.src.proto ipv4, this column specifies the destination IPv4 address in ARP packets. The same limitations apply: ARP traffic is protocol-specific and not representative of broader network behavior [70].

Impact: As with the source ARP field, this column is unlikely to add value unless the IDS is tailored to detect ARP-specific anomalies [70].

#### http.file data.

Reason: This specific column houses the actual file data transmitted over HTTP. Raw packet data is unstructured and bears significant variations, making it difficult for our model to extract meaningful patterns during the training phase. Furthermore, processing raw packet data can be computationally expensive and may require highly domain-specific preprocessing (e.g., malware signature matching) [71].

Impact: Without feature extraction (e.g., file type, size, or entropy), this column is not directly related to IDS model training [71].

#### http.request.fulluri.

Reason: This column bears the full URI of an HTTP request. URIs are highly variable and often contain unique identifiers (e.g., session tokens, query parameters). This variability makes them unsuitable for direct use in training without further processing [71].

Impact: Extracting specific features, such as URI length, query parameter count, or suspicious keywords in the URI, might be more effective [71].

#### icmp.transmit timestamp.

Reason: This column flags the timestamp included in ICMP (Internet Control Message Protocol) packets. Like frame.time, raw packet timestamps are not inherently very useful for detecting intrusions/ attacks unless processed into meaningful time-based features [68].

Impact: ICMP timestamps are barely indicative of any malicious activity on their own [70].

#### http.request.uri.query.

Reason: This column has the query string portion of an HTTP request URI. Query strings are highly variable and often included in unique session or user-specific data, making them unsuitable for direct use in training intrusion detection models [71].

Impact: Further processing is required to extract features such as query length, parameter count, or suspicious patterns (e.g., SQL injection signatures) for this column to contribute to the training process of an IDS model [71].

#### tcp.options.

Reason: This column consists of TCP options, which are optional fields in TCP headers used for advanced functionality (e.g., window scaling, timestamps) to optimize TCP operations. These options are highly technical and vary depending on the protocol implementation, making them less generalizable for IDS training [72].

Impact: Unless the IDS is specifically designed to detect anomalies in TCP options, this field is unlikely to add any value in training of the IDS model [72].

#### tcp.payload.

Reason: This column contains the raw payload of TCP packets. Raw payloads are unstructured and vary widely, making them unsuitable for models to interpret into meaningful training without further processing. Additionally, payloads often contain encrypted data, which cannot be analyzed without decryption [72].

Impact: Feature extraction (e.g., payload size, entropy) or deep packet inspection (DPI) would be required to make this column useful [72] in the training process.

#### tcp.srcport.

Reason: This column stores the source ports of TCP packets. Source ports are often ephemeral and randomly assigned to TCP or UDP packets, making them less informative for detecting attacks [73].

Impact: Instead of raw source ports, analyzing port usage patterns or commonality might be more helpful towards training an AI model [73].

#### tcp.dstport.

Reason: This column has the destination port of TCP packets in this dataset. The destination ports can indicate the type of network service being accessed; they are often well-known and static (e.g., port 80 for HTTP). This limits their usefulness for detecting attacks [73].

Impact: Destination port usage patterns or deviations from expected ports might provide more insight [73].

#### udp.port.

Reason: In our dataset, this column specifies the UDP port number of the UDP packet. Just like TCP ports, UDP ports are also either ephemeral or well-known, which limits their direct contribution to IDS training [73,74].

Impact: Analyzing port usage patterns or anomalies might be more effective than using raw port numbers [73].

#### mqtt.msg.

Reason: This column contains MQTT (Message Queuing Telemetry Transport) messages in the dataset under consideration. MQTT is a lightweight protocol frequently used in IoT devices, where the raw message content is highly variable and context-dependent. Without any prior processing, this column is not directly usable for IDS training [75].

Impact: Feature extraction (e.g., message frequency, size, or topic analysis) would be necessary to make this column meaningful to any model for training [75].

### Data preprocessing

#### Data cleaning.

After pruning the dataset, we implemented a row-wise deletion process for NaN values. Each row with one or more NaN values was deleted. After this, duplicate values are deleted. During this phase, duplicate rows were deleted. Duplicate rows with the first occurrence retained, and subsequent duplicates were removed. The changes were applied directly to the original dataset.

#### Feature encoding and balancing.

Subsequently, we executed the process of one-hot encoding. In this process, categorical variables are converted into a numerical format. One-hot encoding was performed on the following columns as candidates for transformation. 1:‘http.request.method’, 2:’http.referer’, 3:’http.request.version’, 4:’dns.qry.name.len’, 5:’mqtt.conack.flags’, 6:’mqtt.protoname’, and 7:’mqtt.topic’.

### Model training

The Adaptive Moment Estimation (Adam) optimizer with a learning rate of 0.001 was used for model training. This algorithm provides a superset of the advantages ofRMSProp andAdaGrad. The selected learning rate of 0.001 defines the step size of the algorithm taken in each iteration for optimization. It’s worth considering that the learning rate has a considerable impact on the training and the performance of the final model. A very high learning rate may result in overshooting the optimal solution and lead to instability; on the other hand, a very low learning rate may result in slow convergence or getting stuck in local minima. The decision to use a learning rate of 0.001 was reached after a series of experiments to ensure optimal performance.

#### Implementation of training loop.

**Fig 13:**
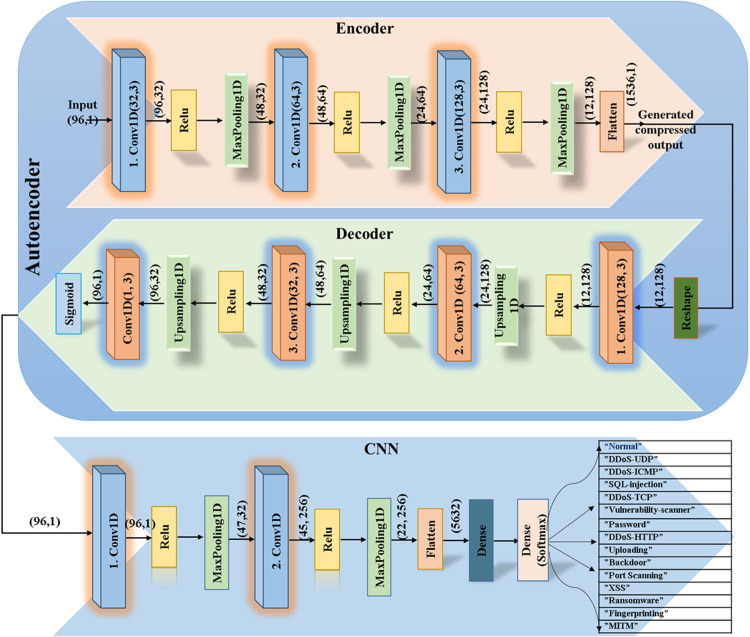
Detailed architecture of ourproposedCNN-based Approach [39].

We used TensorFlow to facilitate model training. The Detailed architecture of the autoencoder (encoder, decoder) and CNN is described above in Fig 13.The following critical parameters were tuned, which were essential for training a CNN:

*Model structural design:* The design of the CNN, including the number of layers and neurons.

*Loss Function:* The function used to measure the model’s performance during training.

*Metrics:* The evaluation metrics to monitor the model’s performance.

*Training Data:* The subset of the dataset used to train the model, including the features and labels columns.

*Validation Data:* A subset of the dataset used to validate the model’s performance after training.

By setting the parameters above, we aimed to create a robust model capable of accurately classifying different types of cyberattacks based on the processed dataset.

**Fig 14:**
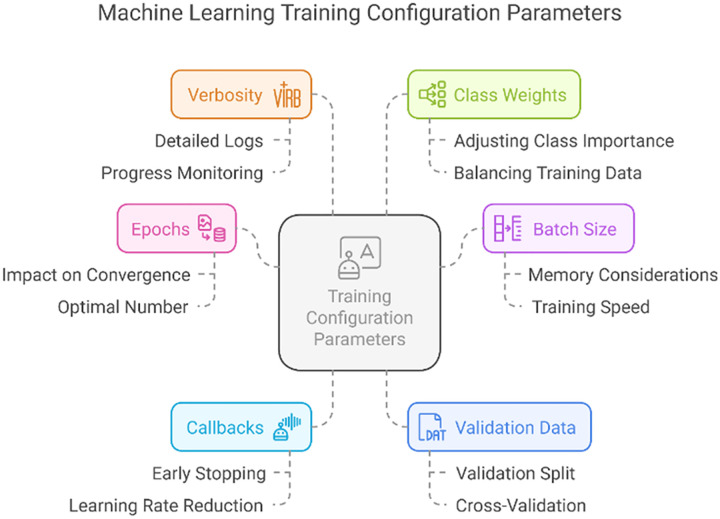
Training process explanation [63].

The training process is explained in Fig 14 above. Here, we outline the key training configuration parameters used in machine learning model training, specifically considering epochs, batch size, callbacks, validation data, verbosity, and class weights. Here is a brief understanding that these parameters arecrucial for optimizing model performance and ensuring efficient training processes.

#### Epochs.

The value of variable EPOCHS = 5 defines the number of training epochs, which indicates how many times the model will iterate over the entire training dataset. All epochs enable the model to learn from previous data, gradually improving its performance.

#### Batch size.

This parameter defines the number of training samples used in each iteration. This parameter has a significant impact on both the memory footprint and training speed. A larger batch size can speed up the training phase but may require more memory, whereas a smaller batch size can lead to more stable updates, albeit at the expense of slower training. We have used the value of 256 for this parameter.

#### Callbacks.

The callbacks parameter includes functions such as early stopping and lr-reduce. Callbacks are functions that can be invoked at different stages during training to perform specific actions. They are essential for monitoring the training process and making intelligent adjustments as needed.

#### Early stopping and Lr-reduce.

Early stopping is a callback function that stops the training process if a monitored metric (such as validation loss) shows no further improvement, thereby preventing overfitting. On the other hand, lr-reduce is used to dynamically adjust the learning rate during the training phase for better convergence.

#### Validation data and validation split.

The parameter Validation Split = 0.2 specifies the size of the validation dataset, or the percentage of the training data to reserve for validating the performance. This plays a crucial role in evaluating the model’s performance on unseen data.

#### Verbose.

This parameter determines the level of loquaciousness during the training phase. The value set to 1 typically delivers progress updates for all epochs, thus allowing us to monitor the training process in real time.

#### Class weight.

The class weight parameter is an optional argument whose value can be set to provide weights to classes if our dataset is not balanced. This supports the model in placing more weight on understated classes, resulting in improved overall performance on imbalanced datasets.

It is significant to note that we programmed our code to include early stopping and lr-reduce callbacks. Proper configuration of these parameters is essential for practical model training and achieving optimal results.

### Parameters configuration

We are describing the test/train split function, which divides our dataset into test and training sets. This function is used explicitly in ML to break the dataset into two further subsets: one is reserved for training of the model, and the other for testing and performance evaluation of the model. A brief explanation of the parameters under consideration in our experiments is described below:

#### test size = 0.2.

This parameter postulates the percentage of the dataset that must be assigned for testing. In our experiment, we have fixed it to 0.2; as a result, 20% of the dataset will be reserved for testing.

#### random state = 1.

The reproducibility of a random dataset is ensured by setting it to 1; in this way, we guarantee that the exact split will be generated each time we execute the code with the same dataset, using the same random seed.

#### stratify.

This parameter ensures the identical distribution of classes in the test/train sets, as well as in the original dataset. Now, at this train/ test split step, we shall have the following four arrays:

X-train: Label of training set features.

X-test: Label of testing set features.

Y-train: Label of training set features.

Y-test: Label of testing set features.

At this stage, we use this split dataset in our training and testing loops, respectively. Later, by conducting multiple experiments, we optimized parameters such as batch size, learning rate, and other hyperparameters based on our typical environment and dataset characteristics to achievethe best training/ testing performance. Various performance metrics are considered to provide a precise evaluation of our model’s performance from multiple perspectives. Each metric elucidates a specific aspect of the model’s performance. Keeping an eye on the problem domain and explicit goals, numerous metrics can shed light on trivial insights that serve as a guiding star towards the best model optimization.

### Performance evaluation metrics

There are five evaluation metrics used to accurately express the results obtained by the proposed model: recall, F1-score, accuracy, precision, and ROC-AUC. The graphical depiction of a classification model’s performance can be expressed by an ROC curve at multiple classification thresholds. At the same time, AUC can be used to measure the whole two-dimensional region under the entire ROC curve.

**Fig 15:**
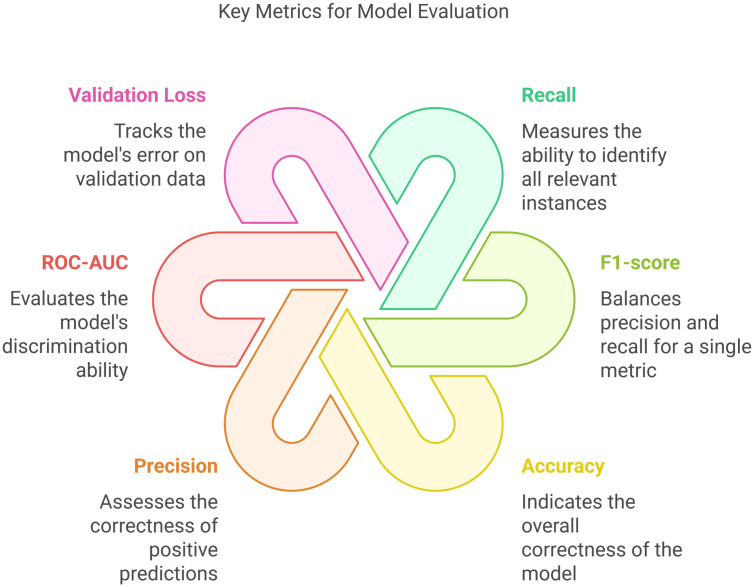
Explanation of performance evaluation matrices [63].

In this section, we outline the key performance evaluation metrics used to measure the effectiveness of the proposed classification model, as portrayed in Fig 15 above. It covers five essential metrics: recall, F1 Score, accuracy, precision, and ROC AUC. Each metric plays a crucial role in understanding the model’s performance and its ability to generalize to unseen data. Additionally, the document discusses validation loss and categorical cross-entropy, which are crucial for evaluating model performance during both the training and validation phases.

#### Accuracy.

Accuracy is a fundamental measure of how many predictions made by the proposed model are correct. It is calculated using the following formula-4:


$$Accuracy=\frac{\left\{TP+TN\right\}}{\left\{TP+TN+FP+FN\right\}}$$ (4)

Where:

TP = True Positives

TN = True Negatives

FP = False Positives

FN = False Negatives

#### Validation loss.

Validation Loss is the loss calculated on the validation dataset that is separate from the training dataset. This metric is crucial for evaluating the model’s performance on unseen data and for detecting overfitting. The formula for validation loss is the same as the loss function associated with the specific problem being addressed. The validation loss can be computed as follows using formula 5:


$$ValLoss=H(p,q)=-\sum {\mathrm{\backslash nolimits}}_{x\in \text{X}}p(x)\text{log}(q(x))$$ (5)

#### Categorical cross entropy CCE.

CCE is particularly useful in Multi-Class Classification problems. It is calculated using the formula 6:


CCE=−∑y_truelog(y_pred) (6)

Where:

(y_true) is the true label

(y_pred) is the predicted probability

#### Recall.

Recall measures a classification model’s abilityto identify relevant data points accurately. It is defined as formula 7:


$$Recall\;=\frac{TP}{TP+FN}$$ (7)

Where:

TP = True Positives

FN = False Negatives

#### F1 score.

The F1 score is a measure of a classification model’s accuracy that considers both precision and recall. It is the harmonic mean of precision and recall, ranging from 0 to 1, where 1 represents the best possible value. The F1 score is calculated as follows in formula 8:


$$F\text{1}=\text{2}\times \;\frac{Precision\times \;Recall}{Precision+Recall}=\frac{\text{2}\;xTP}{\text{2}\;x\left\{TP+FP+FN\right\}}$$ (8)

#### ROC curve and AUC.

The Receiver Operating Characteristic (ROC) curve is a graphical representation of a classification model’s performance across various classification thresholds. The Area Under the Curve (AUC) represents the entire two-dimensional area under the ROC curve, providing a single measure of overall model performance. We can conclude that these performance evaluation metrics are essential for estimating the strengths and weaknesses of a classification model, guiding improvements, and ensuring robust performance on unseen data.

## Results and discussion

During the experimentation phase, we conducted a series of eight experiments. These experiments were performed with various parameters on the same dataset. The results of all experiments are described in Table 4. The parametric variations affecting the output values, such as accuracy and loss, are described in the table below. To facilitate the analysis, the results are also visualized through various graphs to support the findings presented in the same table, allowing for the identification of high-performing experiments with optimal results. However, as we examine the series of output parameters, it becomes apparent that various parameters are optimized in different experiments. As described in Table 4, the proposed DLGAN-based IDS technique outperforms in terms of accuracy, processing time and validation loss. The DLGAN based IDS also achieves a higher detection rate and a lower false positive rate. If we focus on our old experiment-0 [39], in its 2nd EPOCH we observed best processing time (processing time = 703s along with processing time per epoch = 112ms) with a decent validation loss (val loss = 0.1491), later on in second epoch the accuracy was further increased to 94% but at the cost of higher processing time that is 1033 sec. The table provided summarizes the results of six different experiments out of a set of eight experiments (Exp-0 to Exp-8) conducted to train a model, having a neural network, under varying conditions. Below is a breakdown of the key components and observations from the table.

**Table 4:** All experiments and readings.

	Exp-0 [39]	Exp-1	Exp-2	Exp-3	Exp5	Exp8
**Training Accuracy (Epoch 1)**	92.27%	86.91%	84.06%	90.74%	91.14%	89.96%
**Validation Accuracy (Epoch 1)**	92%	94.18%	92.50%	95.17%	95.12%	95.96%
**Training Loss (Epoch 1)**	0.1583	0.3351	0.3984	0.2418	0.23	0.266
**Validation Loss (Epoch 1)**	0.1491	0.1329	0.1795	0.1036	0.0972	0.095
Processing time (Epoch 1) sec*1000	0.703	0.535	0.537	0.508	0.566	0.625
**Training Accuracy (Epoch 2)**	94%	94.20%	92.51%	95.26%	95.47%	95.64%
**Validation Accuracy (Epoch 2)**	94%	94.42%	92.96%	95.69%	95.72%	95.83%
**Training Loss (Epoch 2)**	0.1087	0.1289	0.1755	0.1039	0.0987	0.095
**Validation Loss (Epoch 2)**	0.1087	0.1213	0.1619	0.0904	0.1001	0.0939
Processing time (Epoch 2) sec*1000	1.033	0.527	0.544	0.468	0.515	0.601
**Optimizer**	Adam	Adam	Adam	Adam	Adam	Adamax
**Learning rate**	0.001	0.001	0.001	0.001	0.001	0.001
**Dense**	64	64	64	64	256	256
**Remarks**	Old Exp with over sampling	Exp with GANs and 50000 Oversampling	Exp with GANs and 70000 Oversampling	Exp with GANs	Exp with GANs having 256 Dense	Exp with GANs having 256 Dense, Adamax optimizer

### Experiments (Exp-0 To Exp-8)

Each experiment represents a different configuration or approach to training the model. The experiments differ in terms of oversampling techniques, optimizers, dense layer sizes, and other hyperparameters.

### Metrics

Training Accuracy: The observed accuracy of the model on the training dataset during each epoch.

Validation Accuracy: The accuracy of the model on the validation dataset during each epoch after training.

Training Loss: The value of loss (e.g., cross-entropy loss) on the training dataset during training in each epoch.

Validation Loss: The loss value on the validation dataset during each epoch.

Processing Time: The time taken to compute/ process one epoch, measured in seconds (s).

### Hyperparameters

Optimizer: We found Adam or Adamaxto be the best performers, which are optimization algorithms used to update the model’s weights.

Learning Rate: After trying other values (0.01, 0.002, 0.0001) we fixed it at 0.001 for all experiments.

Dense Layer Size: The number of neurons in the dense (fully connected) layer of the model. We experimented its variations between 64 and 256 to record the results.

### Remarks

Here in this row, we describe the specific configuration or technique that were used in each experiment, such as oversampling, use of GANs (Generative Adversarial Networks), or changes in the size of Dense layer and optimizer.

### Observations

**Fig 16:**
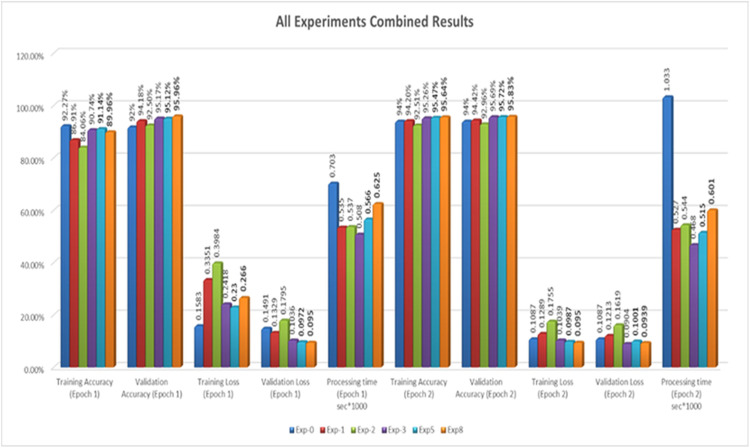
All experiments’ graphical depiction..

**Fig 17:**
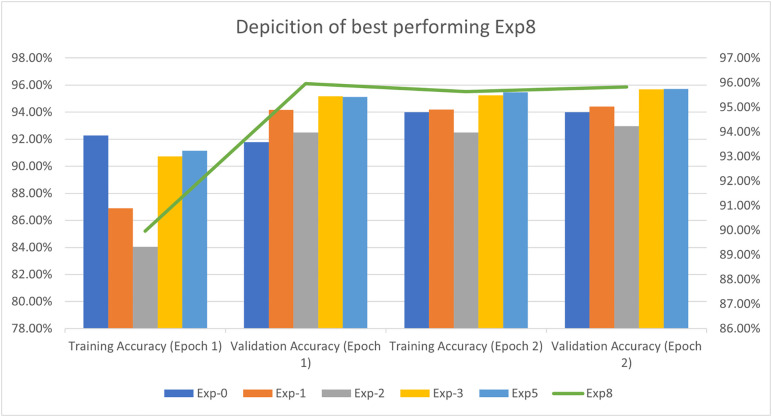
Explanation of best performing Exp8 among all experiment.

**Fig 18:**
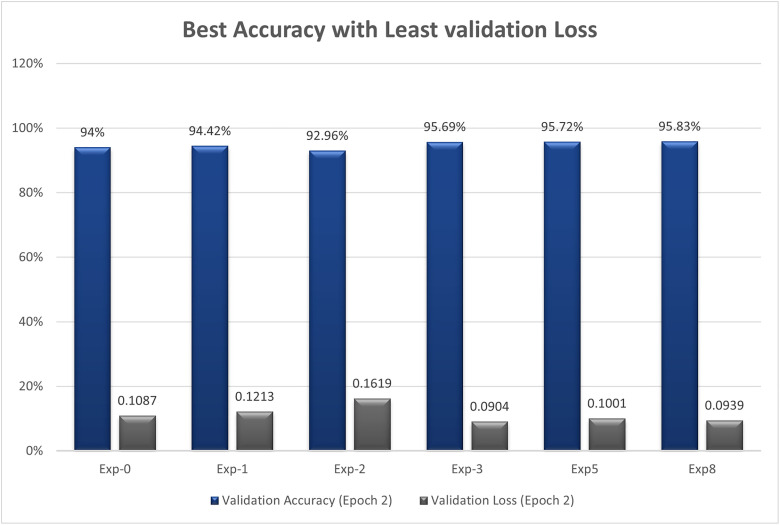
Explanation of best accuracy and least validation loss among all experiments.

The following observations are deduced from a series of experimental results depicted in Figs 16–18.

### Training and validation accuracy

#### Exp-0 (Old Exp [39] with oversampling).

Achieves high training accuracy (92.27% in Epoch 1, 94% in Epoch 2) but slightly lower validation accuracy (92% in Epoch 1, 94% in Epoch 2). It is interpreted that this model generalizes well but may have some overfitting.

#### Exp-1 and Exp-2 (GANs with oversampling).

These experiments have lower training accuracy in Epoch 1 (86.91% and 84.06%) compared to Exp 0, but higher validation accuracy (94.18% and 92.50%). However, as we pass through Epoch-2, training accuracy improves significantly (from 94.20% to 92.51%), and validation accuracy remains high (from 94.42% to 92.96%). This indicates that GANs with oversampling have contributed to improved generalization.

#### Exp-3, Exp-5, and Exp-8 (GANs with larger dense layers and Adamax optimizer).

These experiments exhibit consistently higher training and validation accuracy across both epochs. Exp-8 (with Adamax optimizer) achieves the highest validation accuracy (95.96% in Epoch 1, 95.83% in Epoch 2).

### Training and validation loss

#### Exp-0.

This experiment depicts moderate training and validation loss values, indicating decent performance as mentioned in [39].

#### Exp-1 and Exp-2.

These two experiments have higher training loss in Epoch 1, but lower validation loss, suggesting better generalization.

#### Exp-3, Exp-5, and Exp-8.

This set of three experiments consistently exhibits low training and validation loss values, indicating excellent model performance and generalization.

#### Processing time.

Exp-0 has the highest processing time, likely due to the older configuration. Exp-3 has the lowest processing time, suggesting that it is the most efficient in terms of computational expensiveness.

### Optimizer and dense layer size

#### Adam vs. adamax.

Adamax optimizer used in Exp-8 shows slightly better validation accuracy and lower validation loss as compared to Adam in Exp-5, suggesting that it may be more effective for this task.

#### Dense layer size.

Larger dense layers size (256 neurons in Exp-5 and Exp-8) seems to improve performance compared to smaller layers (64 neurons in Exp-0 to Exp-3).

### Key notes

#### 1. GANswith oversampling.

As per our core idea the use of GANs for oversampling to balance out our dataset (Exp-1 to Exp-8) improves validation accuracy and reduces over-fitting as compared to traditional oversampling (Exp-0).

#### 2. Larger dense layers.

It is evident in our experiments that increasing the number of neurons of Dense Layer, from 64 to 256 improves our model performance, as seen in Exp-5 and Exp-8.

#### 3. Adamax optimizer.

During experimentation, it was found that Adamax (Exp-8) outperforms Adam in terms of validation accuracy and loss, suggesting that it is a better choice for this task.

#### 4. Efficiency.

**Fig 19:**
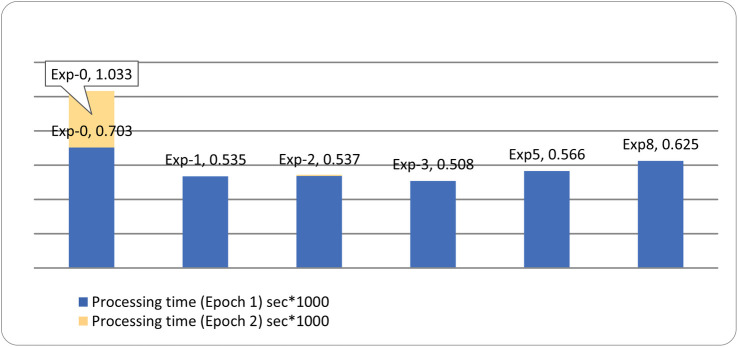
Processing time of each epoch in all experiments.

Out of a series of eight experiments, we found that Exp-3 is the most computationally efficient, with the lowest processing time of 508 seconds, as depicted in Fig 19.

#### 5. Best experiment.

Out of all experiments, Exp-8 stands out as the best-performing experiment, achieving the highest validation accuracy (95.96% in Epoch 1 and 95.83% in Epoch 2) and the lowest validation loss (0.095 in Epoch 1 and 0.0939 in Epoch 2).

#### 6. Recommendations.

The bird’s-eye view of all experiments enables us to utilize GANs to balance the dataset, which is more effective than oversampling and a larger dense layer (256 neurons) for improved performance. We recommend considering using the Adamax optimizer instead of the Adam optimizer for improved validation accuracy. If computational efficiency is a priority, Exp-3 is a good choice, as it strikes a balance between performance and processing time.

## Conclusions

We propose an architecture with two improvements in this paper, one of which is DLGAN for balancing the dataset of IoT devices. The second is to harness the power of HPC as the devices are connected to an HPC cloud via satellite link. These two modifications enhance the security of IoT devices. The proposed system can provide intrusion detection with better accuracy as the technique is deployed on a central HPC-enabled cloud server. To achieve this purpose, we utilized a publicly available real-life dataset. We trained our model on it using DL and balanced the dataset using DLGAN, and then assessed the performance of the complete proposed architecture. The proposed architecture is recommended for deployment to enhance the integrity and security of IoT networks in reallife and mission-critical environments. It exhibits the potential to utilize AI and DL based approaches to enhance the efficacy of Intrusion Detection Techniques (IDTs), which have been emphasized as a means to improve our architecture, as mentioned in this paper. We also explored the challenges and other factors involved in fortifying IoT sensors/ devices with satellite links.

Our proposed infrastructure, in conjunction with IDT, can detect both malicious and benign traffic with improved accuracy. It is capable of detecting 14 types of CS threats by leveraging the power of AI-based algorithms, such as CNN and autoencoders. Our proposed architecture is first trained and then detects 14 different threats in real-time. By having this capability, the proposed IDT consecutively monitors devices’ network activity and satellite Ethernet network data while residing in the central HPC-enabled cloud.

The resource-hungry nature of DL algorithms is optimized to minimize the consumption of computational resources while maintaining strong attack detection capabilities. During our research, we also examined the specific characteristics of satellite network data, including inconsistent connectivity, low bandwidth, and latency. The analysis based on experimental evidence sheds light on the processing hunger of the adopted ML algorithms and their reliance on tunable hyperparameters, in addition to which the smooth operation is justified by considering Ethernet as a communication protocol in satellite-based IoT networks. The IDT’s integration with the satellite network becomes feasible with the aforementioned consideration in our architecture.

Efficacious adoption of the DLGANs to generate samples to balance our dataset requires considering hyperparameters, which are explained in section Hyperparameter Tuning. These measures help address the requirement of enhancing the capabilities/ accuracy of our set of ML algorithms to detect attacks and vulnerabilities, ensuring that the IDT continues to mitigate security risks in the future.

As the adoption of IoT devices increases by leaps and bounds, driving the increased adoption of satellite connectivity, research and development, along with AI-enabled Intrusion Detection Techniques for IoT sensors/devices connected using satellite links, will be an utmost requirement in the future. This architecture exhibits a promising contemporary security framework to fortify IoT satellite networks against 14 types of attacks. Finally, improvements in the security posture of IoT deployments with satellite connectivity in their last-mile connections to IoT sensors.

## Future directions

Upon reviewing the latest literature, it becomes apparent that GANs have demonstrated superior performance in various applications across nearly every field. Therefore, we have adopted it to enhance the security of satellite-based IoT networks. The GANs-based framework comprises a generator that generates new data and a discriminator that validates these instances. This dynamic facilitates self-improvement of effective learning and tracing of patterns, which can play a pivotal role in detecting and mitigating potential security threats in satellite communications and IoT infrastructures, as mentioned in [76,77]. During case studies, it is evident that the intersection of deep learning, cybersecurity, and IoT illustrates the emerging landscape of technology,serving as a guiding star for future research and development to create novel applications.

Future work in line with our experiments includes exploring the scalability and robustness of our proposed architecture in bigger and mission-critical IoT environments. Broaden the spawn by adding more sensor data in the dataset, and adding more types of vulnerabilities/ attacks/ threats in our dataset. Dataset prepared by capturing packets from a network having a satellite link at its last-mile edge, IoT node. Future research directions also include further optimization of AI algorithms. Identifying more security measures to be tailored around large-scale IoT deployments, and quantifying scalability challenges in worldwide deployment architecture.

The landscape of satellite-based IoT networks is undergoing rapid transformation, particularly with the integration of innovative technologies such as deep learning and GANs. The adoption of deep learning methodologies, particularly models like DLGAN, offers promising advancements in attack detection and data security, which are very critical, especially in the context of satellite communications and IoT applications [19,49]. It can be used at the core of the attack detection engine in the future.

### Enhanced security protocols.

One of the core areas of emphasis will be the development/ deployment of enhanced security protocols, futuristically tailored around the satellite IoT networks. As the day-to-day reliance on interconnected devices increases in the future, so does the threat surface, with attackers increasingly targeting vulnerabilities in IoT devices [16,17]. In future research, we should emphasize creating adaptive security frameworks/ architectures that leverage the intelligence of machine learning algorithms to detect and deter potential attacks in real-time, thereby guarding sensitive IoT data [78].

### Integration of edge computing.

Another important future direction is the integration of edge computing within satellite IoT networks, by processing data closer to the source at the last mile of the network, rather than burdening the centralized cloud systems. In this way, edge computing can significantly reduce latency and improve the efficiency of data handling as suggested in [46]. This shift will also enhance the security posture differently by transmitting less data over potentially insecure networks, resulting in a reduction of the risk of interception and unauthorized access, as discussed in [16].

### Response to anomalies.

As soon as the system detects an unusual occurrence, it raises an alert. However, these alerts must be handled with care. Fully automated responses without human oversight can lead to false alarms or unintended consequences. Therefore, a dashboard is recommended in the future so that any action taken in response to a detected anomaly should be logged and displayed promptly, and, where possible, reviewed by a human operator.

### AI and machine learning advancements.

The widespread use of artificial intelligence (AI) and machine learning (ML) will play a pivotal role in analyzing vast amounts of data generated by satellite IoT devices. Sophisticated analytical techniques powered by AI can provide predicted, actionable insights, allowing us to perform predictive deterrence/ maintenance, along with improved decision-making processes, in various applications, such as smart cities and autonomous vehicles [46]. The emerging evolution of AI technologies will pave the way for developing futuristic instruments to tackle the complexities associated with IoT security and data management [16].

### Collaborations and standardization.

Future advancements will also leverage the potential for increased collaboration among various stakeholders, including technology giants, government management authorities, and industry leaders. The establishment of futuristic, standardized protocols for satellite IoT security will be indispensable in creating a cohesive framework that ensures interoperability and enhances overall system resilience against sophisticated threats, as mentioned in both [78,79]. Moreover, documenting the secure deployment guidelines and benchmarks for security practices will also facilitate a more uniform approach across the board in managing the diverse range of devices within satellite networks [15].
