# Correction: A GAN-Based Approach for enhancing security in satellite based IoT networks using MPI enabled HPC

**DOI:** 10.1371/journal.pone.0339335

**Published:** 2025-12-18

**Authors:** Syed Zubair Ahmad, Farhan Qamar, Hamdan Alshehri, Fathe Jeribi, Ali Tahir, Shams Tabrez Siddiqui, Jayabrabu Ramakrishnan

There is an error in affiliation 2 for authors Hamdan Alshehri, Fathe Jeribi, Ali Tahir, Shams Tabrez Siddiqui and Jayabrabu Ramakrishnan. The correct affiliation 2 is: Department of Computer Science, College of Engineering and Computer Science, Jazan University, Jazan 45142, Saudi Arabia.

## References

[1] Ahmad SZ, Qamar F, Alshehri H, Jeribi F, Tahir A, Siddiqui ST, et al. A GAN-Based Approach for enhancing security in satellite based IoT networks using MPI enabled HPC. PLoS One. 2025;20(9):e0331019. doi: 10.1371/journal.pone.0331019 40997099 PMC12463295

